# The tropical cookbook: Termite diet and phylogenetics—Over geographical origin—Drive the microbiome and functional genetic structure of nests

**DOI:** 10.3389/fmicb.2023.1089525

**Published:** 2023-03-14

**Authors:** Juan José González Plaza, Jaromír Hradecký

**Affiliations:** Faculty of Forestry and Wood Sciences, Czech University of Life Sciences Prague, Prague, Czechia

**Keywords:** metabolomics, metabarcoding, metagenomic sequencing, termite nest microbiome, termite diet, phylogenetic relatedness

## Abstract

Termites are key decomposers of dead plant material involved in the organic matter recycling process in warm terrestrial ecosystems. Due to their prominent role as urban pests of timber, research efforts have been directed toward biocontrol strategies aimed to use pathogens in their nest. However, one of the most fascinating aspects of termites is their defense strategies that prevent the growth of detrimental microbiological strains in their nests. One of the controlling factors is the nest allied microbiome. Understanding how allied microbial strains protect termites from pathogen load could provide us with an enhanced repertoire for fighting antimicrobial-resistant strains or mining for genes for bioremediation purposes. However, a necessary first step is to characterize these microbial communities. To gain a deeper understanding of the termite nest microbiome, we used a multi-omics approach for dissecting the nest microbiome in a wide range of termite species. These cover several feeding habits and three geographical locations on two tropical sides of the Atlantic Ocean known to host hyper-diverse communities. Our experimental approach included untargeted volatile metabolomics, targeted evaluation of volatile naphthalene, a taxonomical profile for bacteria and fungi through amplicon sequencing, and further diving into the genetic repertoire through a metagenomic sequencing approach. Naphthalene was present in species belonging to the genera *Nasutitermes* and *Cubitermes*. We investigated the apparent differences in terms of bacterial community structure and discovered that feeding habits and phylogenetic relatedness had a greater influence than geographical location. The phylogenetic relatedness among nests' hosts influences primarily bacterial communities, while diet influences fungi. Finally, our metagenomic analysis revealed that the gene content provided both soil-feeding genera with similar functional profiles, while the wood-feeding genus showed a different one. Our results indicate that the nest functional profile is largely influenced by diet and phylogenetic relatedness, irrespective of geographical location.

## 1. Introduction

Termites represent one of the most interesting stories of adaptation in the evolution of the animal kingdom since they obtain their energetic resources from lignocellulose, a recalcitrant material. The use of lignocellulosic sources depends largely on the symbiotic relationships with a wide range of microorganisms (Brune, 1998; Ohkuma and Brune, 2010). Termites interact with these microorganisms for energetic purposes but are also deeply related to them because they live on their surfaces, in their nest material, or in their food substrates. Nests are the Achilles' heel of termites since their whole social structure revolves around their central place of existence. That is so because termites live in populated colonies with a series of intricate galleries where the conditions of humidity (Wiltz et al., 1998) and temperature create an ideal setting for the growth of pathogenic organisms such as fungi. Not exclusively because of these two, but also due to the high content of nutrients in termite nests in comparison with the nutrient-poor surrounding soil environment (López-Hernández, 2001; van der Sande et al., 2018), yet rich in nutrient cycling (Jordan and Herrera, 2015). Among the reasons for the high content of nutrients in nests and their immediate surrounding areas is the way in which nests are built. Nests are a product of a considerable amount of feces as a cementing tool (Jose et al., 1989; Chouvenc et al., 2018) and soil particles that are transported from the surrounding environment (Mizumoto et al., 2021). Considering these limited availabilities of food substrates in the surrounding soil, the conditions are set for a competitive race for survival in the microbial moiety, with a continuous input of external strains potentially detrimental and threatening the existence of the whole colony. These external microorganisms thrive in an environment that could limit the presence of grazing organisms (Clarholm, 1981; Rønn et al., 2002), which also has a high availability of nutrients provided by the input of termite feces when they maintain and build their nests (Chouvenc et al., 2013). However, termites are very successful in their strategies for limiting the growth of detrimental microorganisms, which is an important aspect of their ecological success. Among them, it has been established that fumigation with certain types of compounds, such as naphthalene, aids in controlling pathogens (Chen et al., 1998a; Wright et al., 2000). The role of the nest microbiome in maintaining a pathogen-free environment is not clear, though studies have addressed its potential (Chouvenc et al., 2018).

In this context, we carried out a study to better characterize the microbial communities living in termite nests among several termite species in several geographical locations. Before generalizing into how the microbiome takes part in the defense, we need to understand which factors drive communities into one composition or another. The defense strategies may vary according to the input of nutrients that they receive (termite diet), the lifestyle of the termite (genus as a product of the evolutionary history that determined the termite's interaction with the environment), or what type of microbial communities and ecological features exist (represented by the geographical origin of the termite). Our questions aim to determine the metabolite, taxonomic, and metagenomic composition. We hypothesize that the nutrient input plays an important role and that the microbiome will show a different pattern regardless of the geographical origin.

We sampled nests from termites with two feeding strategies, namely, wood and soil, originating on both sides of the Atlantic, including rainforest and savanna representatives.

To evaluate the presence of volatile compounds, we used a coupled untargeted gas chromatography-mass spectrometry (GC-MS) analyte identification strategy, followed by a targeted naphthalene analysis. These metabolomics approaches were complemented with a taxonomic characterization of the microbial communities existing on the same material to try to understand the potential co-occurrences of certain identified metabolites with bacterial or fungal genera. Finally, we further performed shotgun metagenomic sequencing from selected termite colonies for a deeper understanding of the genomic diversity of nests and what types of genes could be involved in biocontrol strategies or, to a certain extent, in microbiological warfare.

Our results showed a much lower amount of naphthalene in termite nests than those previously reported in the literature. A very interesting observation found throughout the three omics approaches (metabolomic, amplicon sequencing, and metagenomic sequencing) is that termite genera and especially the feeding habits determine the relationships between the different microbial communities, where bacterial communities are mostly affected by host phylogenetic relatedness and fungi by diet, with a lower impact of the geographical origin for both cases.

## 2. Material and methods

### 2.1. Sample collection and initial processing

Nests from 28 termite colonies were collected during 2019 and 2020 at different sampling campaigns in South America and Africa (Table 1). The first sampling site is located in French Guiana, Petit-Saut Dam area, which is located in the Sinnamary River (5°03′ N, −53°02.76′ E), and is characterized by hot and humid weather throughout the year with two dry seasons (February to March and July to November) and two rainy seasons (Colas et al., 2020).

**Table 1:** Species identification after COII method.

**Genus**	**Species**	**Feeding habits**	**Origin**	**Termite feeding group**	**Code**	**Sampling campaign**	**Accession (16S)**	**Accession (ITS)**	**Accession (shotgun)**
*Coptotermes*	*formosanus*	Wood	BR	I	Copfor BR I	July 2019	ERX9702657	ERX9703253	
*Nasutitermes*	sp.	Wood	BR	II	Nassp BR II	July 2019	ERX9702672	ERX9703268	
*Nasutitermes*	*lujae*	Wood	CM	II	Naslu 1 CM II	July 2019	ERX9702667	ERX9703263	ERX9702718
*Nasutitermes*	*lujae*	Wood	CM	II	Naslu 2 CM II	July 2019	ERX9702668	ERX9703264	ERX9702719
*asutitermes*	*lujae*	Wood	CM	II	Naslu 3 CM II	July 2019	ERX9702669	ERX9703265	ERX9702720
*Nasutitermes*	sp.	Wood	CM	II	Nassp CM II	July 2019	ERX9702673	ERX9703269	ERX9702721
*Microcerotermes*	sp.	Wood	CM	II	Micsp CM II	January 2020	ERX9702666	ERX9703262	
*Cephalotermes*	*rectangularis*	Wood	CM	II	Cefre CM II	July 2019	ERX9702655	ERX9703251	
*Constrictotermes*	*cavifrons*	Lichens	FG	II	Conca FG II	January 2020	ERX9702656	ERX9703252	
*Nasutitermes*	*similis*	Wood	FG	II	Nassi 1 FG II	May 2019	ERX9702670	ERX9703266	
*Nasutitermes*	*similis*	Wood	FG	II	Nassi 2 FG II	May 2019	ERX9702671	ERX9703267	
*Anoplotermes*	*banksii*	Soil	FG	III	Anba 1 FG III	May 2019	ERX9703247	ERX9702651	
*Anoplotermes*	*banksii*	Soil	FG	III	Anba 2 FG III	May 2019	ERX9702652	ERX9703248	ERX9702711
*Anoplotermes*	*banksii*	Soil	FG	III	Anba 3 FG III	May 2019	ERX9702653	ERX9703249	ERX9702712
*Anoplotermes*	*banksii*	Soil	FG	III	Anba 4 FG III	May 2019	ERX9702654	ERX9703250	ERX9702713
*Embiratermes*	*neotenicus*	Soil	FG	III	Embne FG III	January 2020	ERX9702664	ERX9703260	
*Neocapritermes*	*taracua*	Soil	FG	III	Neota 1 FG III	January 2019	ERX9702675	ERX9703271	
*Neocapritermes*	*taracua*	Soil	FG	III	Neota 2 FG III	May 2019	ERX9702676	ERX9703272	
*Neocapritermes*	*taracua*	Soil	FG	III	Neota 3 FG III	May 2019	ERX9702677	ERX9703273	
*Silvestritermes*	*heyeri*	Soil	FG	III	Silvhe FG III	January 2020	ERX9702678	ERX9703274	
*Termes*	*fatalis*	Soil	FG	III	Terfa FG III	January 2020	ERX9702679	ERX9703275	
*Cubitermes*	*planifrons*	Soil	CM	IV	Cubpl CM IV	February 2020	ERX9702661	ERX9703257	ERX9702714
*Cubitermes*	*sulcifrons*	Soil	CM	IV	Cubsu CM IV	February 2020	ERX9702662	ERX9703258	ERX9702715
*Labiotermes*	*labralis*	Soil	FG	IV	Labla FG IV	January 2020	ERX9702665	ERX9703261	
*Cubitermes*	*aff. ugandensis*	Soil	MW	IV	Cubug MW IV	July 2019	ERX9702663	ERX9703259	
*Cubitermes*	*inclitus*	Soil	MW	IV	Cubin MW IV	July 2019	ERX9702658	ERX9703254	ERX9702716
*Cubitermes*	*muneris*	Soil	MW	IV	Cubmu 1 MW IV	July 2019	ERX9702659	ERX9703255	ERX9702717
*Cubitermes*	*muneris*	Soil	MW	IV	Cubmu 2 MW IV	July 2019	ERX9702660	ERX9703256	

The feeding habits of termites, their geographical origin, and classification according to the termite-feeding group are shown as well. For metagenomic data analysis after sequencing, we have used the generic Ban1, Ban2, and Ban3 for simplicity purposes and have the following correspondence: Ban1 = Anba 2 FG III, Ban2 = Anba 3 FG III, and Ban3 = Anba 4 FG III. Breed samples were collected in our termite collection at the Czech University of Life Sciences, Prague, Czech Republic.

N/A, not applicable; BR, breeds; CM, Cameroon; FG, French Guiana; MW, Malawi. The sequencing data have been deposited in the ENA archive under project accession PRJEB55779.

In Africa, we collected samples in Ebogo village (Cameroon, 3°23.86'N, 11°28.19'E), located in the Mbalmayo Forest Reserve. This area is characterized by a bimodal precipitation pattern (Knoben et al., 2019), with a dense evergreen forest subjected to perturbations on approximately a third of the territory due to agricultural practices (Mey and Gore, 2021). The dry periods span from December to February and July to August (Zapfack and Engwald, 2008). The second African sampling site was Nyika National Park (Malawi, −10°32.92'N, 33°53.47'E), characterized mostly by miombo woodland, and to a lesser extent, montane dambos and grasslands, with mean average temperatures of 23°C (Allingham and Harvey, 2013), and a dry season that lasts mostly from June to October, with rainfalls occur chiefly during November to March (van Velden et al., 2020). Two of the samples originate in the breeds kept at the Faculty of Forestry and Wood Sciences (Czech University of Life Sciences Prague, Czech Republic) at humid constant temperatures of 28°C.

Nest samples were processed in clean, hexane-wiped metal trays under a laminar flow hood to avoid contamination. Large pieces of nests devoid of termites were broken into small fragments and stored at −80°C until processing. Samples were further homogenized in a Retsch oscillatory mill. The hexane-wiped steel 10-ml chambers were filled with nest material, sealed, and submerged in liquid nitrogen until stabilized. Samples contained in steel chambers were then homogenized to a fine powder for 5 min at 30 Hz frequency, recovered, and stored at −80°C until further analysis.

### 2.2. Metabolite profiling

For initial headspace solid-phase microextraction (HS-SPME) volatiles profiling, 200 mg of previously homogenized samples were placed into different 10-ml screw-top vials for headspace analysis and were sealed with a magnetic cap. Sample incubation and the following metabolite extraction were performed at 50°C in the agitator of the Gerstel MPS2 autosampler (Gerstel, SUI) for 10 and 30 min, respectively. For separation and detection, a gas chromatograph coupled with a time-of-flight mass spectrometer (GC-TOF-MS; Leco Pegasus 4D, Leco, USA) was used. The temperature-programmed injector was operated in splitless mode at 275°C. For separation, a 30-m (0.25 mm i.d., 0.25 μm film thickness) Rxi-5Sil MS (Restec, USA) column was used. The temperature program for the 1D oven was as follows: 40°C for 1 min, then ramped at a rate of 10°C/min to 210°C, and finally at 20°C/min to 300°C with a hold time of 3 min. The total GC run time was 26 min. The mass spectrometer was operated in the mass range of 35–500 m/z with an acquisition speed of 10 Hz.

Peak find, mass spectral deconvolution, and peak alignment were performed in ChromaTOF software (LECO, USA). Compounds with an automatically selected quantification mass and a signal-to-noise ratio (S/N) higher than 50 were selected for alignment. The mass spectral similarity of signals in different samples, to be considered as the same compound, must be higher than 60% and the retention time difference must be lower than 5 s. A tentative identification based on the spectral similarity of the deconvoluted spectrum with spectra stored in the NIST mass spectral library (NIST17) was provided for each aligned compound. Before statistical analysis, data were normalized using constant row sum (each signal quantification mass area in one sample is divided by the sum of all signal quantification areas in the respective sample) and then transformed using the centered log-ratio (clr) transformation. All samples were analyzed using principal component analysis (PCA) to find the separation and grouping of samples, while only samples with more than three representatives per genus were processed *via* partial least squares discriminant analysis (PLS-DA). Based on the variable importance plot (VIP) in PLS-DA models, interesting compounds underwent further confirmation of identity *via* comparison of the calculated retention index with retention indices from the NIST lib.

For targeted naphthalene analysis, an SPME method based on Tsimeli et al. (2008) and Cao (2012) was used, employing standard addition quantification. In total, 100 mg of homogenized sample were placed into a 10-ml HS vial, and 0.5-ml of water (UHPLC-MS purity, Supelco, USA) was added before sealing with a magnetic screw cap. Four vials per sample were prepared. A volume of 10 μl of methanol (HPLC Plus purity, Sigma-Aldrich, USA) was injected through the septum into the first two vials, while 10 μl of methanol containing different amounts of naphthalene was injected into the remaining two vials. The sample was heated under agitation at 50°C for 10 min, and then volatiles from the headspace were collected onto an SPME fiber for 5 min.

A linear response of naphthalene was observed when a blank nest sample was spiked at levels between 1 and 1,100 ng/g. Method accuracy and reproducibility were checked by measuring a certified reference material (CRM) of polyaromatic hydrocarbons in soil (CRM170, Lot: LRAC8900, Sigma-Aldrich, USA). Measuring six replications of CRM, the relative standard deviation (RSD) of the entire determination was 12%, resulting in an uncertainty (U = 2 × RSD) of 25%. Comparing obtained naphthalene content of 563 ng/g ± 140 ng/g (average ±U) to the certified value of 573 ± 45 ng/g showed good accuracy of the presented method. The only modification for CRM measurement was that due to the high naphthalene concentration, the sample weight was reduced 10-fold to 10 mg per vial.

### 2.3. Species identification

DNA from five termite workers belonging to each colony was isolated using the Qiagen Blood and Tissue Kit according to the manufacturer's instructions. Isolated DNA was used for amplification of the cytochrome oxidase II gene (COII) with the following program: 1 min at 94°C, 30 cycles of 94°C for 15 s, 61°C for 1 min, 72°C for 35 s, and a final step of elongation at 72°C for 10 min. PCR products were submitted to Microsynth AG (Switzerland) for Sanger sequencing. Results were compared to the NCBI nr database for retrieving BLAST results (Altschul et al., 1990), where the result with the highest identity percentage was selected. The sequencing primers were previously described by Benjamino and Graf (2016).

### 2.4. Microbial DNA isolation

Powder-homogenized samples were used for the isolation of DNA. A quantity of 100 mg of soil was used as starting material using the NucleoSpin Soil DNA Kit (Macherey-Nagel, Germany). The whole procedure was carried out under laminar flow using sterile material, and kit components were exclusively opened and manipulated under sterile conditions. Samples were quantified by a Qubit 2.0 Fluorometer (Thermo Scientific, USA), and quality was checked through spectrophotometric methods.

### 2.5. Sequencing

All sequencing was carried out following standard company procedures (Novogene, Hong Kong, China). Amplicon sequencing was carried out on all samples, while shotgun metagenomic sequencing was performed on selected samples (both indicated in Table 1).

#### 2.5.1. 16S rRNA gene sequencing

Primers 515F (GTGCCAGCMGCCGCGGTAA) and 806R (GGACTACHVGGGTWTCTAAT) were used to sequence the 16S V4 region. In brief, a 292-bp amplicon was obtained through PCR with the above-mentioned specific primers. PCR products were quantified, purified (Qiagen Gel Extraction Kit, Qiagen, Germany), and used for library preparation (NEBNext Ultra DNA Library Pre-Kit, Illumina, San Diego, CA, USA). Libraries were sequenced following a paired-end 250-bp sequencing strategy on NovaSeq 6000 Illumina instruments.

#### 2.5.2. Internal transcribed spacer (ITS) sequencing

In brief, 1 ng of DNA was used to amplify fungal ITS genes from the ITS2 region with a specific set of primers, namely, ITS3 (GCATCGATGAAGAACGCAGC) and ITS4 (TCCTCCGCTTATTGATATGC), with an amplicon size of 386 bp. The overall procedure with amplicons follows the one explained for 16S.

#### 2.5.3. Metagenomic sequencing

We selected two genera, comprising four samples each, from those where naphthalene was produced (*Cubitermes* and *Nasutitermes*). For comparison purposes, a third group with no naphthalene presence in nests was selected (*Anoplotermes*). After quality control in standard agarose gels and quantity concentration estimation with Qubit 2.0 (ThermoFisher, USA), DNA was randomly sheared using sonication. Fragments (150 bp) were end-polished, A-tailed, and then the Illumina adapters (5' adapter: 5'-AGATCGGAAGAGCGTCGTGTAGGGAAAGAGTGT-3'; 3' adapter: 5'-GATCGGAAGAGCACACGTCTGAACTCCAGTCAC-3') were ligated. PCR amplification was carried out using P5- and P7-indexed oligonucleotides and products were purified with the AMPure XP system. The size distribution of libraries was evaluated using the Agilent 2100 Bioanalyzer (Agilent Technologies, CA, USA) and quantified using real-time PCR for achieving equimolar concentrations. Sequencing (paired-end) was subsequently carried out on NovaSeq 6000 Illumina instruments.

### 2.6. Microbiome data processing and analysis

#### 2.6.1. Amplicon sequencing data: 16S and ITS

The assignment of paired-end reads to samples was based on their unique barcodes, which were later truncated by cutting off the barcode and primer sequence. Overlaps of paired-end reads served for merging reads using FLASH (Magoč and Salzberg, 2011). Reads were quality filtered to obtain high-quality clean tags (Bokulich et al., 2013) using QIIME (V1.7.0). Reads were then compared to a reference database (Gold DB, http://drive5.com/uchime/uchime_download.html) with the UCHIME algorithm (Edgar et al., 2011) for the detection and removal of chimera sequences. Clean reads were stored in individual files in fastq format. These files were used as input in a common tool used in microbiome studies for the bioinformatic treatment of clean, demultiplexed reads, QIIME2 (release 2021.4; Bolyen et al., 2019). This wrap-up tool was used to import (q2-import, SingleEndFastqManifestPhred33V2) the clean demultiplexed single fastq files corresponding to our samples with the import plugin. Denoising was carried out with DADA2 (*via* q2-dada2-denoise single; Callahan et al., 2016), which allows for the identification of all observed amplicon sequence variants. ASVs were aligned using mafft (Katoh et al., 2002; *via* q2-alignment) and used to build a phylogeny (fasttree2), *via* q2-phylogeny (Price et al., 2010).

After DADA2, sequences that were found in the negative control “.fastq” file were removed from the project through quality-control exclude-seqs plugins.

Core metrics were calculated with the core-metrics-phylogenetic plugin, where rarefaction was performed using a sampling depth of 10,000 for 16S data. Among metrics, we calculated Faith's phylogenetic diversity (Faith, 1992), beta diversity such as UniFrac (Lozupone et al., 2007), unweighted UniFrac (Lozupone and Knight, 2005), Jaccard distance, and Bray-Curtis dissimilarity. We analyzed the microbial communities in the nests with a quick overview of the bacterial community compositions through the calculation of the Bray-Curtis dissimilarity metric (Sorensen, 1948; Bray and Curtis, 1957). This index provides an idea of similarity/dissimilarity due to the product of present taxa and their relative abundances (van Rensburg et al., 2015). The ACE index of α-diversity provides an overview of the microbial richness within one sample, without considering the ASVs abundance (Qiao et al., 2017). At β-diversity, the weighted UniFrac distance can represent the differences between samples according to the evolutionary history (Lozupone and Knight, 2005; Lozupone et al., 2007, 2011), and how different are two microbial communities. Plugins for 16S amplicon data were core-metrics-phylogenetic, alpha-group-significance, and beta-group-significance, where the metadata column of choice was “geography,” “genus,” or “feeding-group.” We calculated non-phylogenetic “ace” diversity for ITS data with the “diversity alpha” plugin and a beta diversity through “diversity beta” (“Bray-Curtis”). Significance was tested through the “diversity beta-group-significance” (ANOSIM test).

Taxonomy was assigned with the q2-feature classifier classifysklearn naïve Bayes taxonomy classifier (Bokulich et al., 2018; Kaehler et al., 2019); for the 16S, we used the weighted Greengenes 13_8 99% OTUs full-length sequences (DeSantis et al., 2006), and for the ITS, we used UNITE (https://doi.plutof.ut.ee/doi/10.15156/BIO/2483915). Taxonomy plots were drawn in R through the ampvis2 package, using the tutorials available at https://sites.google.com/a/ciad.mx/bioinformatica/home/metagenomica/visualizacion/ampvis2 and https://madsalbertsen.github.io/ampvis2/articles/ampvis2.html, where we used the Heillinger transformation in order to represent the PCA (Legendre and Gallagher, 2001). We used the ASV annotated data in R through the ampvis2 package to create PCA plots.

Once taxonomy was assigned and frequency was calculated per sample, we performed a least discriminant analysis (LDA) effect size (LEfSe; Segata et al., 2011). This algorithm discovers biomarkers that identify features of the tested biological conditions. A text-tab-separated file in metadata-columnar format was loaded at a Galaxy instance (https://huttenhower.sph.harvard.edu/galaxy/). In the case of 16S data, the OTU table was filtered to include only those that satisfied frequency > 0.001 in at least one of the samples.

#### 2.6.2. Metagenomics bioinformatics

Sequenced reads were first cleaned using the Trimmomatic (Bolger et al., 2014) and then subjected to a quality control process (FASTX Toolkit 0.0.14. FASTQ/A short-reads pre-processing tools). The Kalamazoo Protocol for metagenome assembly, khmer 0.8.4 (Brown et al., 2013), was then used. We used the digital normalization protocol as stated (Brown et al., 2012, 2015) and used the normalized reads as input for SqueezeMeta v1.4.0, v. May 2021 (Tamames and Puente-Sánchez, 2019), in order to carry out a first assembly using Megahit (Li et al., 2015). We carried out that first assembly using the *seqmerge* mode but did not use the statistics or any other results. After carrying out the assembly, the generated contigs were used as external assembly references (ext-assembly) in SqueezeMeta, and thus, no assembly was performed at this second step. The input for this second round was trimmed and quality control passed, and (in-house script) files had no spaces and contained separate forward and reverse pairs, gzipped. The pipeline followed the usual procedure for SqueezeMeta, with the following tools.

Assembly was carried out with Megahit (Li et al., 2015), contig statistics with Prinseq (Schmieder and Edwards, 2011), and redundant contig removal by CD-HIT (Schmieder and Edwards, 2011). The merging of contigs was carried out with Minimus2 (Treangen et al., 2011), RNA prediction with Barrnap (Seemann, 2014), taxonomy classification of 16S rRNA sequences by the RDP classifier (Wang et al., 2007), tRNA/tmRNA prediction by Aragorn (Laslett and Canback, 2004), and Prodigal for open reading frames (ORF) prediction (Hyatt et al., 2010). Diamond (Buchfink et al., 2014) served as the tool to search for similar patterns at GenBank (Clark et al., 2016), eggNOG (Huerta-Cepas et al., 2016), and KEGG (Kanehisa and Goto, 2000). Then HMM homology searches were performed by HMMER3 (Eddy, 2011) for the Pfam database (Finn et al., 2016). Reads were mapped against contigs with Bowtie2 (Langmead and Salzberg, 2012), and binning was done using MaxBin2 (Wu et al., 2016) and Metabat2 (Kang et al., 2019), where binning results were combined using the DAS Tool (Sieber et al., 2018). MiniPath (Ye and Doak, 2009) was used against the KEGG (Kanehisa and Goto, 2000) and MetaCyc (Caspi et al., 2018) databases for pathway prediction. If not indicated, databases were accepted by default in the SqueezeMeta pipeline.

At the end of the pipeline, tables for results processing in R were created by means of the smq2tables.py script provided in the SqueezeMeta package. Results were loaded in R through the use of SQMtools (Puente-Sánchez et al., 2020), and charts were produced according to the SQMtools manual. Some of the R packages used were “SQMtools,” “ggplot2,” “reshape2,” “pathview,” and “data.table.”

Non-metric multidimensional scaling (NMDS) was carried out in RStudio (RStudio Team, 2020), using Transcripts per Million (TPM) calculated values obtained from the SqueezeMeta pipeline for those genes with KEGG annotation, through the “vegan” package. This ordination collapses the information from all genes vs. the samples to visualize the relationships between samples. The PFAM annotation was interrogated for retrieving functions (through the grep command) and annotated with the following keywords: “antibiotic,” “cellulose,” “phenol,” or “resistance.” TPM values were recorded, and bar plots were built to obtain a quick comparison for those annotated genes among samples.

We further used diamond (Buchfink et al., 2014) in order to search for genes and patterns of interest in our final contig list with Open Reading Frames, where the databases of reference were AnHyDeg (Callaghan and Wawrik, 2016), AromaDeg (Duarte et al., 2014), BacMet (Pal et al., 2014), bactibase (Hammami et al., 2010), CARD (protein homology model; Alcock et al., 2020), and CAZy (Carbohydrate Active enZYmes database, http://www.cazy.org/; Lombard et al., 2014). We used by default an e-value threshold of 0.005, a minimum identity of ≥40%, and manually curated the results, discarding hits with a bit-score of < 30. An arbitrary threshold of ≥90% identity was selected first for considering genes as known, while genes below this threshold were considered novel.

We evaluated the overall differences among samples in terms of the presence of genes (“differentially present genes”) through DESeq2 (Love et al., 2014), according to the methodology explained in the SURFBIO transcriptomics module (https://surfbio.eu/training_wet_dry/), where a gene was considered differentially present when it fulfilled the following conditions: fold change (FC) ≥ 2 or |log2FoldChange| ≥ 1; DESeq2 p-adj ≤ 0.05.

## 3. Results

### 3.1. Identification of termite species belonging to each collected colony

Freshly killed termites belonging to each of the collected nests used in this study were taxonomically identified through molecular methods (COII amplification and sequencing). Results are shown in Table 1. Sequences from this project have been uploaded to the ENA public repository with ref. no. PRJEB55779.

We classified the nests according to the termite species inhabiting them, and only one colony belonged to Group I. Other 10 colonies were classified in Group II from three locations, namely, our laboratory breeds (BR), Cameroon (CM), and French Guiana (FG). Another group comprising 10 colonies originating from French Guiana was classified as Group III. Finally, Group IV comprised colonies originating from Cameroon, French Guiana, and Malawi (MW; Table 1).

### 3.2. Untargeted metabolite profiling

**Figure 1:**
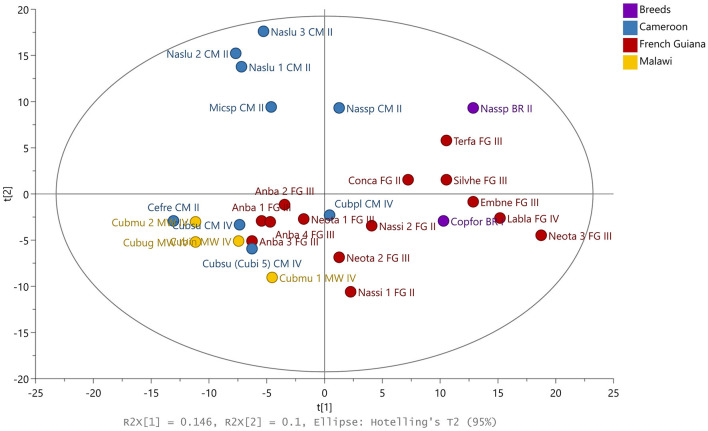
PCA scores plot using SPME-GC-TOF-MS data from the non-targeted analysis. Samples are colored according to geographical location: violet, Breeds; blue, Cameroon; red, French Guiana; yellow, Malawi. Anba, *Anoplotermes banksi*; Cefre, *Cephalotermes rectangularis*; Conca, *Constrictotermes cavifrons*; Copfor, *Coptotermes formosanus*; Cubug, *Cubitermes* aff. *ugandensis*; Cubin, *Cubitermes inclitus*; Cubmu, *Cubitermes muneris*; Cubpl, *Cubitermes planifrons*; Cubsu, *Cubitermes sulcifrons*; Embne, *Embiratermes neotenicus*; Labla, *Labiotermes labralis*; Micsp, *Microcerotermes* sp.; Naslu, *Nasutitermes lujae*; Nassi, *Nasutitermes similis*; Nassp*, Nasutitermes* sp.; Neota, *Neocapritermes taracua*; Silvhe, *Silvestritermes heyeri*; Terfa, *Termes fatalis*; CM, Cameroon; FG, French Guiana; MW, Malawi; I, Feeding Group I; II, Feeding Group II; III, Feeding Group III; IV, Feeding Group IV.

Among the wood-feeding representatives, we observe a clearer separation in the genus *Nasutitermes* between CM and FG (Figure 1). Three samples belonging to the nests of the same species (Naslu 1-3) were grouped together and located in the same quadrant as another CM wood feeder nest (Nassp CM II). The samples originating from MW were grouped together with close to four nest samples belonging to the soil-feeding species (*Anoplotermes banksi*) from FG, along with most of the representatives from feeding groups III and IV.

According to the PLS-DA (Supplementary Figure 1A), a clear separation exists between nests from soil or wood feeders, with influence from genera (Supplementary Figure 1B) and geographical location (Supplementary Figure 1C).

The most responsible volatiles for the separation were used as a class in the PLS-DA models (Supplementary Figure 2), which also used genera [R2X(cum) 0.53, R2Y(cum) 0.99, and Q2(cum) 0.65] and geographical location [R2X(cum) 0.25, R2Y (cum) 0.78, and Q2 (cum) 0.5]. Using the variable importance plot (VIP), the 10 most decisive compounds for separations presented in Supplementary Figure 2 are identified in Table 2.

**Table 2:** Ten most decisive analytes from PLS-DA analysis (Supplementary Figures 1, 2) selected according to VIP.

**A (class: diet; scores plot presented on** **Supplementary Figure 1A****)**								
**Primary ID**	**Variable name**	**RT (s)**	**RI exp**	**RI lit**	**VIP**	**VIP SE 2.44693**	**Average soil feeders (%)**	**Average wood feeders (%)**
15	2,3-Butanedione	369	619	612	1.86	1.60	0.04	0.74
18	2-Butanol	375	626	610	2.05	2.05	0.59	0.07
37	2,3-Pentanedione	435	694	696	3.24	1.42	0.02	0.13
97	cis 1-Ethyl-2-methyl-cyclohexane,	641	916	919	1.88	1.67	0.10	0.02
98	Methyl hexanoate	644	919	925	2.23	1.17	0.00	0.03
127	Methyl 2-methyl-3-furancarboxylate	723	998	999	2.63	0.89	0.00	0.02
132	Beta-Phellandrene	734	1,010	1,015	2.08	0.69	0.03	0.11
138	Limonene	756	1,035	1,030	1.83	0.35	1.05	22.41
154	Terpinolene	807	1,093	1,089	2.60	1.16	0.01	1.61
229	Unknown (RI 1495)	1,120	1,495	-	1.80	1.63	0.89	1.88
**B (class: genera; scores plot presented on** **Supplementary Figure 2A****)**								
**ID**	**Variable name**	**RT (s)**	**RI exp**	**RI lit**	**VIP**	**VIP SE 2.44693**	**Average Ban (%)**	**Average Cub (%)**	**Average Nas (%)**	**Average Neo (%)**
15	2,3-Butanedione	369	582	581	1.63	0.69	0.1	0.0	0.7	0.0
37	2,3-Pentanedione	435	694	696	2.04	1.38	0.0	0.0	0.1	0.0
47	Methyl Isobutyl Ketone	473	737	739	1.57	1.80	0.0	0.0	0.0	0.5
81	1-Hexanol	591	865	867	1.59	0.89	2.7	5.6	0.2	0.3
91	1-Ethyl-4-methylcyclohexane	621	895	888	1.91	0.90	0.0	0.2	0.0	0.0
103	Propyl-cyclohexane	663	938	941	1.66	1.21	0.0	0.2	0.0	0.0
114	1-Octen-3-ol	696	971	979	1.70	0.85	6.1	0.1	0.1	0.4
127	Methyl 2-methyl-3-furancarboxylate	723	998	999	1.61	1.49	0.0	0.0	0.0	0.0
154	Terpinolene	807	1,093	1,089	1.70	1.28	0.0	0.0	1.6	0.0
235	δ-Cadinene	1,147	1,537	1,527	1.64	1.18	0.0	0.0	0.1	0.2
**C (class: geographical origin; scores plot presented on** **Supplementary Figure 2B****)**								
**ID**	**Variable name**	**RT (s)**	**RI exp**	**RI lit**	**VIP**	**VIP SE 2.44693**	**Average CM (%)**	**Average FG (%)**	**Average MW (%)**
15	2,3-Butanedione	369	619	612	1.77	2.03	0.7	0.0	0.0
90	2-Butylfuran	617	891	897	1.76	1.99	0.2	0.2	0.0
101	Unknown (RI 931)	656	931	-	2.04	1.91	0.0	1.5	0.4
103	Propylcyclohexane,	663	938	941	1.87	2.15	0.1	0.0	0.3
122	Unknown (RI 988)	713	988	-	1.94	1.60	0.1	0.2	0.1
185	2-Methyl-dodecane,	946	1,255	1,265	1.93	1.20	0.3	0.4	0.0
213	Ledane	1,039	1,375	1,373	2.07	1.66	0.0	0.1	0.0
218	β-Copaene	1,070	1,420	1,420	1.92	1.49	0.0	0.0	0.0
229	Unknown (RI 1495)	1,120	1,495	1,468	1.96	2.23	0.1	2.5	0.2
232	α-Bulnesene	1,136	1,521	1,512	1.73	1.31	0.1	0.7	0.1

Classes used in PLS-DA are (A) diet, (B) genera, and (C) geographical origin.

VIP, variable importance in projection; RT, retention time; RI, retention index; RI exp, measured retention index; lit, literature; VIP SE, VIP standard error; ID, identifier; Ban, Anoplotermes; Cub, Cubitermes; Nas, Nasutitermes; Neo, Neocapritermes; CM, Cameroon; FG, French Guiana; MW, Malawi.

% refers to the average relative abundance in respective classes.

### 3.3. Targeted analysis: Naphthalene content

Naphthalene is a polyaromatic hydrocarbon observed in the volatile profiling during the non-targeted metabolite analysis. The highest relative abundances were recorded in samples from the genus *Nasutitermes, Coptotermes* from BR, and some of the *Cubitermes*.

**Figure 2:**
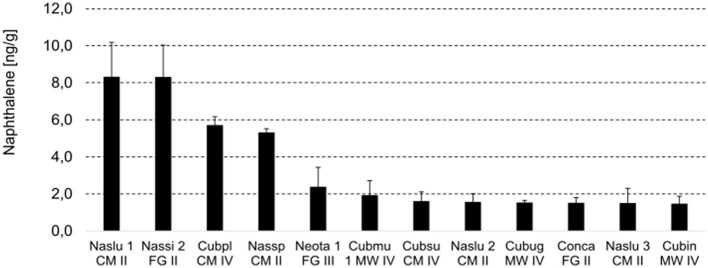
Naphthalene analysis: results from targeted metabolite profiling. Naphthalene concentration is expressed in ng per gram of nest material. Naslu, *Nasutitermes lujae*; Nassi, *Nasutitermes similis*; Cubpl, *Cubitermes planifrons*; Nassp, *Nasutitermes* sp.; Cubmu, *Cubitermes muneris*; Cubsu, *Cubitermes sulcifrons*; Cubug, *Cubitermes* aff. *ugandensis*; Conca, *Constrictotermes cavifrons*; Cubin, *Cubitermes inclitus*; CM, Cameroon; FG, French Guiana; MW, Malawi.

To determine the exact concentration of naphthalene in nest material, we applied a quantitative method, where the concentration ranged from 1.5 to 8 μg/kg (Figure 2). These results indicate that naphthalene is not generally present in the studied species belonging to Group III, except for the nest material from one of the colonies of *Neocapritermes taracua* (colony No. 1). We observed the presence in species belonging to feeding Groups II and IV; in detail, in three samples from the genus *Nasutitermes* (Group II, both Cameroon and French Guiana) and the five species belonging to the genus *Cubitermes* (Group IV). Naphthalene appeared in the colony nest material from *Constrictotermes cavifrons*, belonging to Group II (French Guiana). The samples belonging to Group III do not show detectable amounts of naphthalene (except for one of the *Neocapritermes* samples).

### 3.4. Bacterial community structure

We calculated more than 130,000 effective tags after removing chimera sequences from our samples. These values range from more than 120,000 tags (*Coptotermes*, BR) to more than 140,000 non-chimeric tags (NasLu3, CM). In our DNA isolation process, which was carried out with sterility-opened reagents inside a laminar flow hood throughout the entire isolation process, we included a negative DNA extraction control and yielded more than 36,000 tags after chimera removal.

When comparing the assigned ASVs, 531 belong to the negative extraction control, from a total of 19,753 ASVs in our project. The overlap with other samples was calculated, and from these 532 ASVs, we found to overlap with other samples in a total of 106 ASVs. These relationships are represented as a Venn diagram (Supplementary Figure 3) with selected samples.

Filtering sequences after “dada2” removes the majority of control sequences, leaving a total of 112 tags (from a total of 36,406 before filtering). We observed a decrease in sequences in the rest of the samples, where the minimum number of tags belongs to Neota2FG.III with 38,777 tags.

#### 3.4.1. Subset of samples with at least three representatives per genus

We carried out a sub-selection of samples, as we did not find clear information when using all samples. From a statistical point of view (ACE Index, where the direction is always Group 1 for the first described element and Group 2 for the second), we found that at a Kruskal-Wallis pairwise comparison, there are geographical differences between FG and MW (*p*-value = 0.031), although not significant when taking all groups together (*p*-value = 0.077). Comparing feeding groups, we found significant differences when comparing II vs. III (*p*-value = 0.045) and III vs. IV (*p*-value = 0.015), and these differences are statistically significant among all groups (*p*-value = 0.028). For general, we found differences in *Anoplotermes* vs. *Neocapritermes* (*p*-value = 0.034), *Cubitermes* vs. *Neocapritermes* (*p*-value = 0.02), and *Nasutitermes* vs. *Neocapritermes* (*p*-value = 0.034), existing significant differences among groups (*p*-value = 0.03).

The β-diversity (unweighted UniFrac distance) showed significance between feeding groups (pairwise PerMANOVA; differences between groups II and III, *p*-value = 0.029; groups III and IV, *p*-value = 0.005; differences among all groups, *p*-value = 0.001). In the case of geography, differences were observed between FG and MW (*p*-value = 0.045), while differences among all groups were of *p*-value = 0.02. Data with at least three representatives per genus could be compared by genera as well. In this case, we found statistical differences between *Anoplotermes* and *Cubitermes* (*p*-value = 0.006) and between *Anoplotermes* and *Nasutitermes* (*p*-value = 0.006). Finally, the differences between groups had a *p*-value = 0.001. Further analysis through ANOSIM revealed that all three considered variables (diet, genus, and geographical location) influenced the community following the order (*R*-value): 1 > Genus (0.67) > Diet (0.65) > Geography (0.42) > 0.

**Figure 3:**
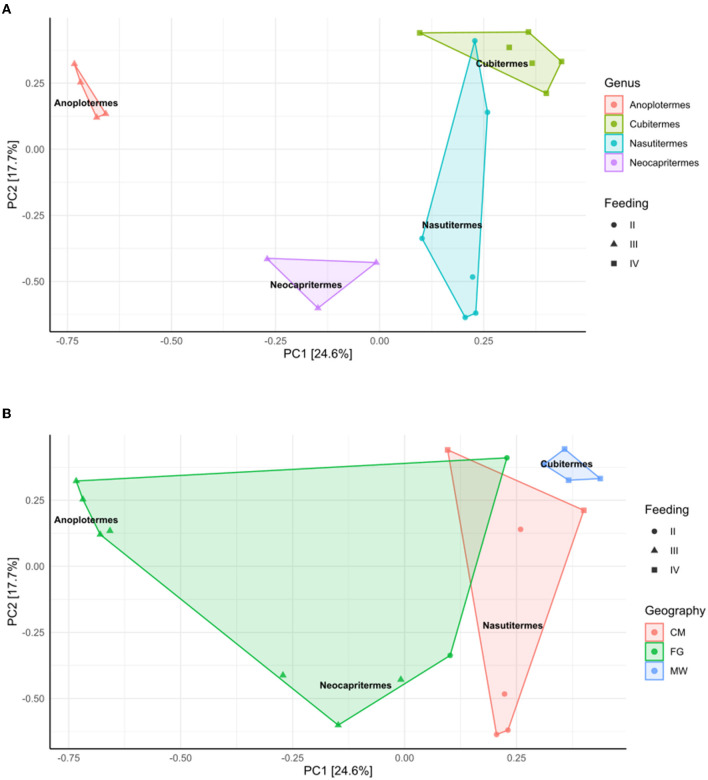
PCA analysis with 16S data after elimination of samples with < 3 representatives per genus, and the tags from the negative sample control. **(A)** Samples were subjected to Hellinger transformation, sample constraint, and color were “Genus,” and shape by feeding strategy. **(B)** Samples were subjected to Hellinger transformation and the sample constraint was “Genus,” while sample color was geography and shaped by feeding strategy. Genera indications are kept as shown in Figure 4.

The PCA plot shows a clear stratification of samples both by genera and diet (Figure 3A), while the stratification is less clear when grouping samples by geographical location (Figure 3B). The two principal components of the analysis explain 24.6 and 17.7% of the variance.

Table 3 shows the phylogenetic assignment of taxa with the most abundant orders, grouping samples by termite genera with a distinction of geographies. We observe an overall representation of *Actinomycetales, Acidimicrobiales, Clostridiales, Gemmatales*, and *Solirubrobacterales*, being *Spirochaetales* well-represented category.

**Table 3:** Taxonomic overview of the samples retained for further analyses (with more than two representatives per genus).

	**Anba (FG)**	**Cub (CM)**	**Cub (MW)**	**NasLu (CM)**	**Nas (FG)**	**NasSp (CM)**	**Neo (FG)**
p_*Actinobacteria*;c__*Actinobacteria*; o_*Actinomycetales*	**32.79**	**39.88**	**31.61**	**37.50**	**24.00**	**44.50**	**34.61**
Unidentified bacteria	**19.22**	**6.42**	**4.69**	**6.13**	**11.55**	3.82	**6.64**
p_*Actinobacteria*;c__*Thermoleophilia*; o_*Solirubrobacterales*	2.35	**5.09**	**5.07**	**7.10**	3.50	4.95	**6.78**
p_*Actinobacteria*;c__*Acidimicrobiia*; o_*Acidimicrobiales*	3.11	**5.87**	3.13	**7.32**	3.21	**9.86**	**8.58**
p_*Firmicutes*;c__*Clostridia*; o_*Clostridiales*	**10.50**	**5.61**	4.98	4.73	**5.60**	**10.46**	2.49
p_*Planctomycetes*;c__*Planctomycetia*; o_*Gemmatales*	2.74	2.66	**6.16**	1.16	**5.26**	0.31	**5.35**
p_*Spirochaetes*;c__*Spirochaetes*; o_*Spirochaetales*	3.07	2.71	1.81	**5.97**	**8.58**	1.79	1.45
p_*Proteobacteria*;c__*Alphaproteobacteria*; o_*Rhodospirillales*	2.80	4.50	3.67	4.73	4.98	3.28	**5.72**
p_*Chloroflexi*;c__*Ktedonobacteria*; o_*Ktedonobacterales*	0.82	0.78	3.48	0.65	0.71	0.00	0.08
p_*Firmicutes*;c__*Bacilli*; o_*Bacillales*	1.19	0.93	1.58	0.81	0.74	1.00	2.76
p_*Proteobacteria*;c__*Alphaproteobacteria*; o_*Sphingomonadales*	0.03	1.33	1.62	1.64	0.66	0.31	0.44
p_*Actinobacteria*	1.02	0.74	0.11	0.07	0.08	0.13	0.10
p_*Proteobacteria*;c__*Alphaproteobacteria*; o_*Rickettsiales*	3.57	1.08	2.13	0.82	4.16	0.11	1.23
p_*Firmicutes*;c__*Bacilli*; o_*Turicibacterales*	1.71	0.87	0.78	0.40	1.14	0.66	1.18
p_*Proteobacteria*;c__*Gammaproteobacteria*; o_*Aeromonadales*	0.00	0.00	0.00	0.00	0.00	2.27	0.00
p_*Chloroflexi*;c__*Ktedonobacteria*; o_*Thermogemmatisporales*	0.08	0.26	1.86	0.06	0.11	0.00	0.21
p_*Proteobacteria*;c__*Betaproteobacteria*; o_*Burkholderiales*	1.39	0.54	1.28	1.38	1.98	0.57	2.81
p_*Acidobacteria*;c__*Acidobacteriia*; o_*Acidobacteriales*	1.50	1.67	2.07	1.16	2.99	0.76	2.36
p_*Firmicutes*;c__*Bacilli*; o_*Lactobacillales*	0.31	0.56	0.42	0.78	0.33	0.61	0.48
p_*Verrucomicrobia*;c__[*Spartobacteria*]; o_[*Chthoniobacterales*]	0.13	0.25	0.63	0.10	0.25	0.01	0.16
p_*Proteobacteria*;c__*Gammaproteobacteria*; o_*Xanthomonadales*	0.27	1.49	1.99	1.80	2.50	0.78	1.56
p_*Proteobacteria*;c__*Alphaproteobacteria*; o_Ellin329	0.23	0.28	0.27	0.22	0.50	0.13	0.49
p_*Synergistetes;c__Synergistia; o_Synergistales*	0.53	0.13	0.18	0.18	0.24	0.09	0.09
p_*Bacteroidetes*;c__*Bacteroidia*; o_*Bacteroidales*	2.13	1.40	1.01	1.13	1.34	1.02	0.33
p_*Acidobacteria*;c__*Solibacteres*; o_*Solibacterales*	0.38	0.73	0.81	0.55	1.40	0.62	0.95
p_*Proteobacteria*;c__*Alphaproteobacteria*; o_*Rhizobiales*	0.19	0.94	1.97	1.63	1.31	0.69	1.91

Taxa with more than 5% representation have been shaded and bolded.

FG, French Guiana; CM, Cameroon; MW, Malawi; Anba, Anoplotermes banksi; Cub, Cubitermes; NasLu, Nasutitermes lujae; Nas, Nasutitermes; NasSp, Nasutitermes sp.; Neo, Neocapritermes taracua.

**Figure 4:**
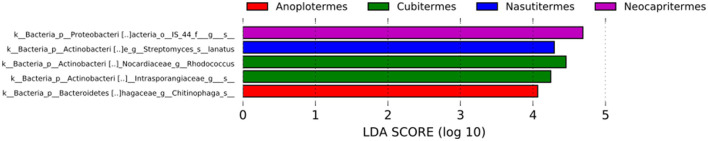
LEfSe results. LDA score (log 10) for each category per genus is shown.

The class Betaproteobacteria, order IS_44, characterized *Neocapritermes* nests in a LEfSe analysis (Figure 4). Representatives from the genus *Streptomyces*, down to species *lanatus*, dominate in *Nasutitermes* nests. *Cubitermes* nests are dominated by members of the genus *Rhodococcus* and the family Intrasporangiaceae, whereas *Anoplotermes* nests are dominated by *Chitinophaga*.

### 3.5. Fungal community structure

The whole group of samples showed inconclusive results; therefore, we analyzed a smaller dataset with more than two representatives per genus. At the alpha diversity by the ACE Index (non-phylogenetic, the direction always from first element to second introduced element), we found only statistical differences among feeding groups at a Kruskal-Wallis pairwise comparison (α ≤ 0.05). Particularly when comparing II vs. III (*p*-value = 0.003), II vs. IV (*p*-value = 0.01), and all groups (*p*-value = 0.006). Further analysis through ANOSIM revealed that all three considered variables (diet, genus, and geographical location) influenced the community following the order (*R*-value): 1 > Diet (0.627) > Genus (0.592) > Geography (0.422) > 0. These results show that fungi are less influenced by the phylogenetic relatedness of the nest host than their bacterial counterparts.

**Figure 5:**
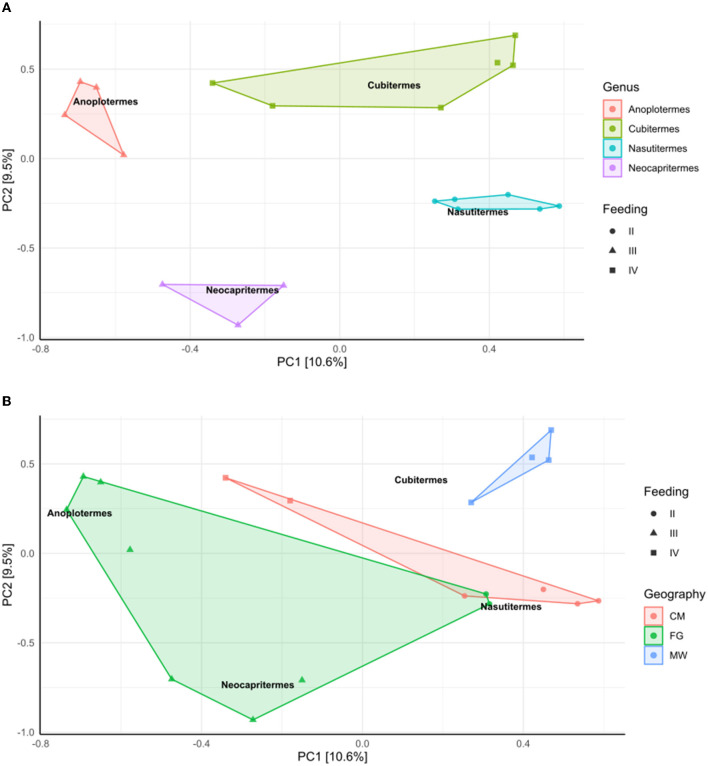
PCA analysis with ITS data after elimination of samples with < 3 representatives per genus, and the tags from the negative sample control. **(A)** Samples were subjected to Hellinger transformation, sample constraint, and color were “Genus,” and shape by feeding strategy. **(B)** Samples were subjected to Hellinger transformation, sample constraint was “Genus,” while sample color was geography, and shape by feeding strategy. Genera indications are kept as shown in Figure 6.

A PCA plot was built in R through the ampvis2 package (Figure 5). Results show a clear stratification of samples, especially by genera, and then feeding strategy (Figure 5A), while the stratification is less clear when grouping samples by geographical location (Figure 5B). The two principal components of the analysis explain 10.6 and 9.5% of the variance.

Taxonomical assignment produced hits mostly to unidentified species, and in this case, we did not proceed with the LEfSe analysis.

### 3.6. Metagenomic sequencing

#### 3.6.1. Cleaning, assembly, and SqueezeMeta pipeline

The first assembly yielded more than 5.5 million contigs, with an average size of more than 720 bp, and N50 of 776 bp.

The reads were assigned taxonomically, and the most abundant hits per sample are shown in Supplementary Table 2, while the statistics on open reading frames (ORFs) are presented in Supplementary Table 3.

#### 3.6.2. Overall characterization of samples

**Figure 6:**
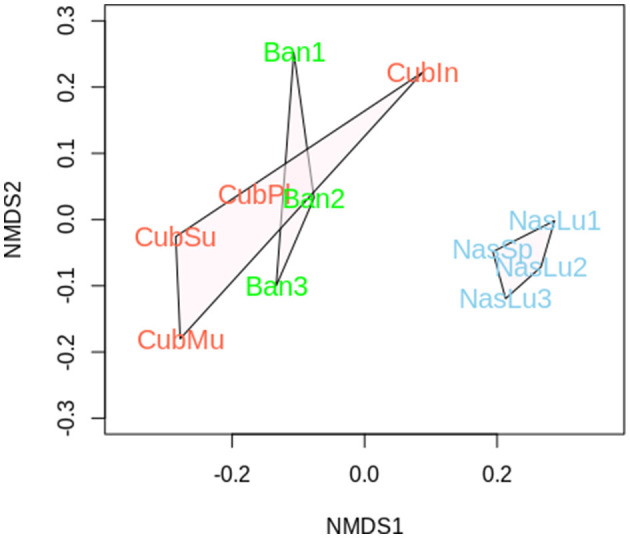
Non-metric multidimensional analysis considering all the samples sequenced in this study (metagenomic shotgun sequencing).

We used the TPM values for all KEGG-annotated genes to evaluate relationships between samples through non-metric multidimensional scaling (NMDS; Figure 6). All *Nasutitermes* samples cluster together, while soil-feeder nests are in another area.

**Figure 7:**
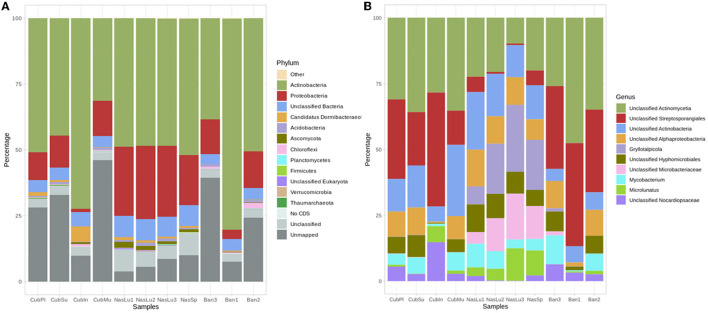
Taxonomic representations obtained through SqueezeMeta pipeline. **(A)** Taxonomic representation at the phylum level. **(B)** Taxonomic representation at the genus level, belonging to the subset “aromatic_aa” (phenylalanine, tyrosine, and tryptophan biosynthesis). Percentages were calculated through SQMtools after SqueezeMeta pipeline.

We can observe that the four samples belonging to the genus *Nasutitermes* (all originating in CAM) have a similar profile at the genus level, while the two soil-feeding genera (*Cubitermes*, from MW and CAM, and *Anoplotermes*, from FG), despite differences at the species level, present a closer profile (Figure 7A). An outstanding exception is a biological replicate Ban1, with a lower representation of Proteobacteria than the rest of samples from this genus (all samples also belong to the species *Anoplotermes banksi*), potentially owed to issues during transportation. For *Nasutitermes*, we find differences between the three *Nasutitermes lujae* replicates and *Nasutitermes* sp. The samples belonging to the genus *Cubitermes* have a more variable profile. Among those, CubPl and CubSu belong to the CM sampling site, while CubIn and CubMu originate in MW.

We further carried out a subset of the present genera belonging to the “aromatic AA” representation (“phenylalanine, tyrosine, and tryptophan biosynthesis”; Figure 7B). The most important genera for soil feeders seem to lie within an unclassified *Streptosporangiales* genus with higher abundance for *Cubitermes* and *Anoplotermes*, while *Nasutitermes* had a higher representation of *Microlunatus*, an unclassified genus within *Microbacteriaceae*, and *Gryllotalpicola*.

We followed a “quick interrogation” step to retrieve from the PFAM-annotated set contigs with the keywords described in the methodology section. We constructed four-bar plots (Supplementary Figures 4, 5) where we observe a different profile between soil and wood feeders. One of the most important categories in *Nasutitermes* seems to be cellulose synthases (Supplementary Figure 4A), while they largely lack some genes annotated as “cadmium resistance transporters” or the “tellurite resistance protein.”

**Figure 8:**
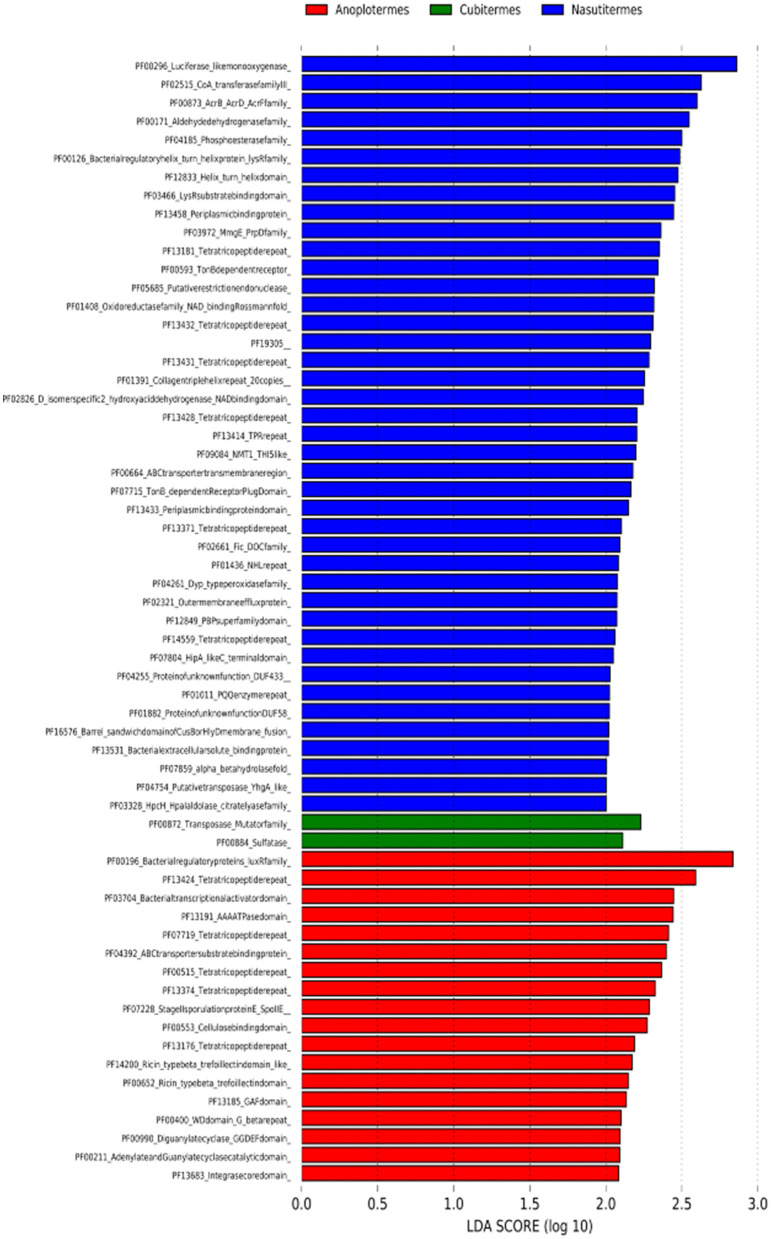
LEfSe analysis for the contigs annotated in PFAM at LDA Score (log_10_) for each category per genus.

An LDA effective size (LEfSe) was calculated using the PFAM annotation with differences among the three represented genera. We obtained 61 classes of annotated genes that are specific biomarkers for each (Figure 8, Supplementary Table 5). *Anoplotermes* was characterized by several biomarkers, among them the first PF00196: bacterial regulatory proteins luxR family, PF13424: tetratricopeptide repeat, PF03704: bacterial transcriptional activator domain, and PF13191: AAA ATPase domain. *Cubitermes* had PF00872: transposases, mutator family, and PF0084: sulfatase as the only biomarker categories found. One of the most represented biomarker categories in the genus *Nasutitermes* was PF00296: luciferase-like monooxygenase, followed by PF02515: CoA transferase family III, PF00873: AcrB/AcrD/AcrF family, and PF00171: aldehyde dehydrogenase family.

#### 3.6.3. Differential gene content analysis

**Figure 9:**
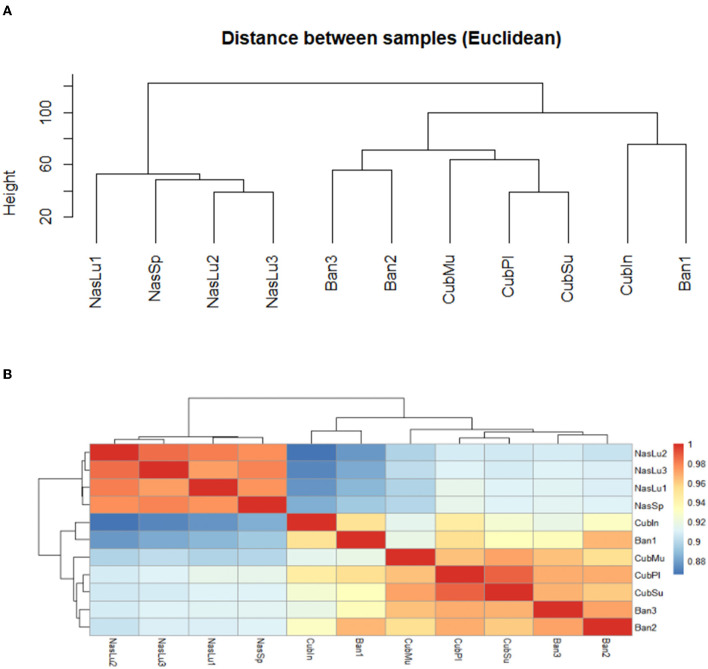
PFAM hierarchical clustering of samples. **(A)** Hierarchical clustering according to Euclidean distance. **(B)** Hierarchical clustering represented by means of a heatmap.

Hierarchical clustering renders two clearly separated nodes (Figure 9A), one with all *Nasutitermes* samples (*Nasutitermes*, CAM) and the other with the soil-feeding genera. In the second group, there are two nodes; one comprises Ban1 and Cubin, and the other is subdivided into *Anoplotermes* (Ban2 and Ban3) and *Cubitermes* samples. Among them, in an inner position, we find the *Cubitermes* samples originating in CAM. These differences can be clearly observed through a correlation heatmap (Figure 9B), while the differentially present genes (DPGs) appear in a PFAM heatmap (Supplementary Figure 6).

The main differences existing between pairs of samples in terms of DPG have been displayed through volcano plots (Supplementary Figure 7). Using COG annotation, at a fold change of 2, we found a total of 2,099 DPGs: 1,147 upregulated genes (more present in *Cubitermes*), when comparing the two soil feeders (*Cubitermes* vs. *Anoplotermes*) and 952 COG annotated genes, with higher presence in *A. banksii* samples (Supplementary Figure 7A). When comparing *Nasutitermes* and *Cubitermes*, we found 6,771 upregulated and 7,871 downregulated DPGs (a statistically significant higher presence in the soil feeder; Supplementary Figure 7B). The last comparison was carried out between *Nasutitermes* and *Anoplotermes* (Supplementary Figure 7C), with 7,152 upregulated and 7,478 downregulated genes.

The functional profiles of soil genera nests had 184 PFAM-annotated contigs with a statistically significant over-presence in *Cubitermes*, while *Anoplotermes* had 265 PFAM-annotated contigs with over-presence. We could equally state that these 265 genes have a decreased presence in the microbial communities from the *Cubitermes* genus, in comparison with *Anoplotermes*. We found some genes with extreme differences in the presence (higher for *Cubitermes*), such as PF03945: delta endotoxin, N-terminal domain (log2FC = 23.96; *p*-adj = 1.55 10^−07^) and PF14470: bacterial PH domain (log2FC = 22.41; *p*-adj = 1.34 10^−15^). On the contrary, PF02183: homeobox-associated leucine zipper (log2FC = −11.78; *p*-adj = 9.10^−14^) has a higher presence in *Anoplotermes*. The comparisons between the microbial communities from the soil-feeding termites with *Nasutitermes'* nest provided again a higher number of DPGs. For *Nasutitermes* vs. *Cubitermes*, we found a total of 1,908 PFAM-annotated contigs with statistically significant over-presence in *Nasutitermes*, while *Cubitermes* had 918 PFAM-annotated contigs with over-presence. We could equally state that these genes have a decreased presence in the microbial communities from *Nasutitermes* than in the microbial communities on those nests from *Cubitermes*. Among the functions most present in *Nasutitermes*, we found PF16937: type III secretion system translocator protein, HrpF (log2FC = 12.16; *p*-adj = 7.20 10^−13^), PF17667: fungal protein kinase, HrpF (log2FC = 10.99; *p*-adj = 2.26 10^−07^), and PF05420: cellulose synthase operon protein C C-terminus (BCSC_C; log2FC= 10.92; *p*-adj = 1.01 10^−15^). For instance, PF03945: delta endotoxin, N-terminal domain (log2FC = −28.16, *p*-adj = 8.66 10^−14^) is an example of a highly present PFAM function in *Cubitermes* (Supplementary Table 6).

The comparison between *Nasutitermes* and *Anoplotermes* yielded 1,962 upregulated and 1,016 downregulated genes (Supplementary Table 6).

#### 3.6.4. Annotation of ORFs in specialized databases

We carried out further annotation of the ORFs retrieved by SqueezeMeta with several specialized databases of interest (see the “Material and Methods” section). The first characterization of these results was to count known genes over a 90% identity threshold. We used a blasting approach with a lower identity threshold of 40% but focused on the bit-score for annotation (especially ≥ 50), based on Pearson (2013), to discover homologous genes. We have used this approach because we found cases where a 100% BLAST identity had a length of alignment under 50 amino acids. Using the maximum bit-score values obtained through DIAMOND Blast (Buchfink et al., 2014), the e-value was zero in 28 hits at the BacMet list, with alignment lengths varying from 605 to 1,118 amino-acids for those 28 genes, and identities ranging from 55 to 99%. As a result, and in accordance with the study by Enault et al. (2016), we suggest that the use of bit-score is a much better indication than identity for discovering homology to described genes. We used the annotation obtained through diamond Blastx with three databases, namely, AromaDeg, CARD, and CAZy. These lists were used as input for differential gene content analyses.

**Figure 10:**
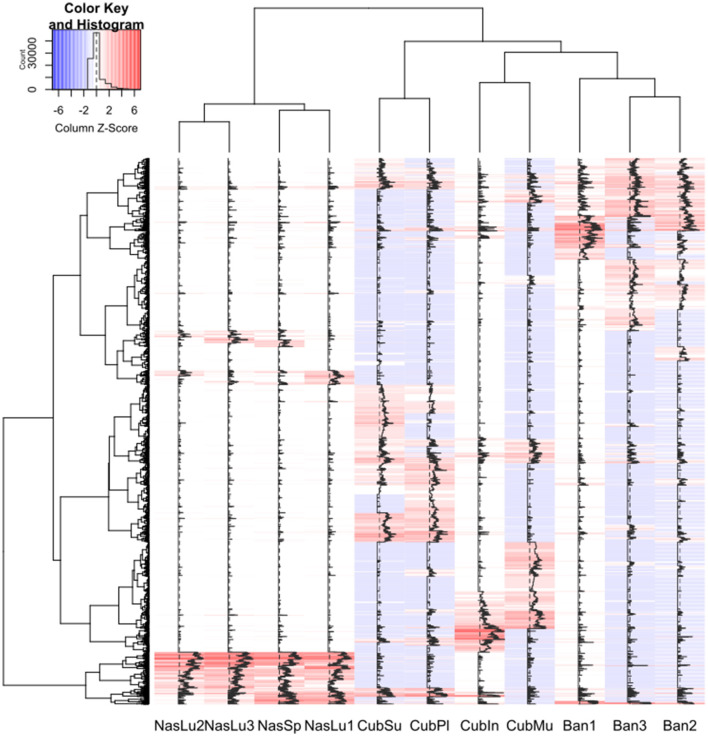
Heatmap of genetic content profiles for the Aromadeg annotation set. Samples or genes clustered using Euclidean distance. Ban, *Anoplotermes banksi*; CubIn, *Cubitermes inclitus*; CubMu, *Cubitermes muneris*; CubPl, *Cubitermes planifrons*; CubSu, *Cubitermes sulcifrons*; NasLu, *Nasutitermes lujae*; Nassp, *Nasutitermes* sp. Numbers after sample name indicate replicate number.

The AromaDeg-annotated genes served as an input for a heatmap displaying the gene content profile in all samples (Figure 10). We found clear differences and tested them for statistical significance. There were 8,132 AromaDeg-annotated genes, from which the comparison of *Cubitermes* vs. *Anoplotermes* yielded 14 upregulated and 21 downregulated DPGs. The comparison of *Nasutitermes* vs. *Cubitermes* was carried out, and 468 genes were upregulated or significantly more present in the *Nasutitermes* genus, while 82 were downregulated or had a higher presence in the *Cubitermes* genus. For the comparison of *Nasutitermes* vs. *Anoplotermes*, we found 224 upregulated and 12 downregulated genes.

The comparison between the soil feeders yielded 54 upregulated genes (*Cubitermes*), and 241 genes had a higher presence in *Anoplotermes*. The correlation among samples and hierarchical clustering clearly divided all samples by genera. The comparison of *Nasutitermes* vs. *Cubitermes* resulted in 1,587 CARD-annotated genes with a higher presence in the wood feeder nest community, while the soil feeder nest community had 988 upregulated genes. When *Nasutitermes* was compared with *Anoplotermes*, we found 1,403 upregulated and 1,745 downregulated genes (higher presence in the soil feeder).

We carried out the analysis with the CAZy DB-annotated genes. In the comparison between both soil feeders, we found no statistical differences. *Nasutitermes* vs. *Cubitermes* yielded 287 upregulated and 2 downregulated genes. We found a larger number of genes for the *Nasutitermes* vs. *Anoplotermes* comparison. This one provided 519 upregulated and 218 downregulated genes.

Some of the most deregulated genes for each of the three analyses are displayed in Table 4.

**Table 4:** Genes with differential presence obtained from the specific annotations with AromaDeg, CARD, and CAZy databases.

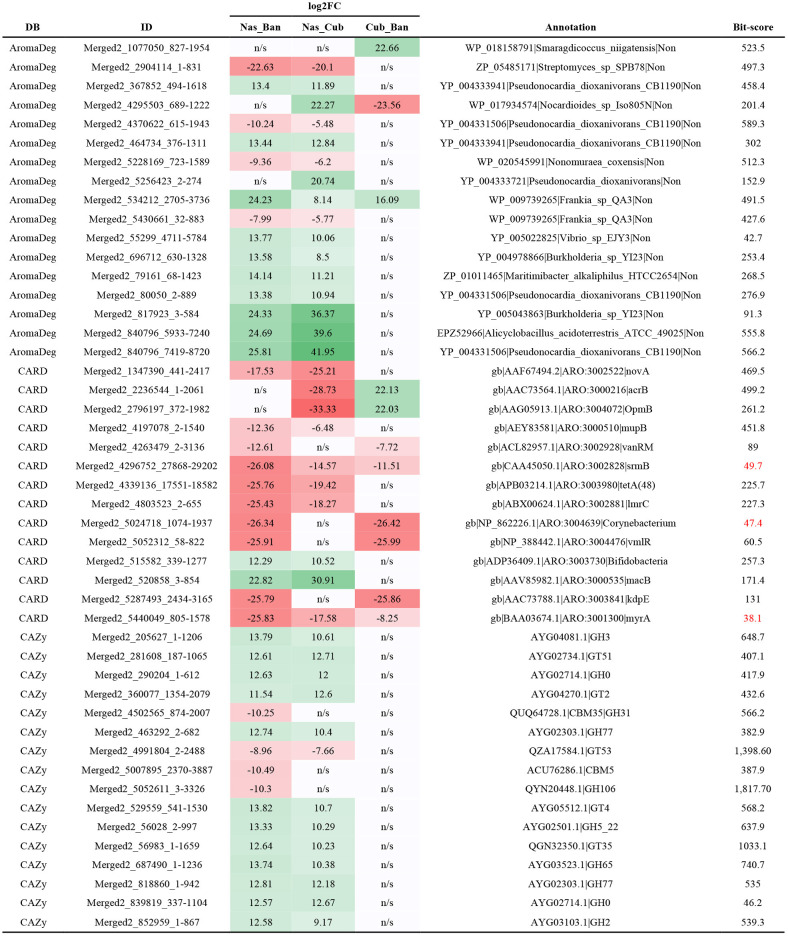

Bit-score values below 50 have been indicated in red-color font. FC, fold change; DB, database; ID, identifier; Nas, *Nasutitermes*; Ban, *Anoplotermes*; Cub, *Cubitermes*; n/s, not significant. Red shading, under-represented or under-expressed genes. Green-shading, over-represented or over-expressed genes.

## 4. Discussion

We evaluated a wide range of termite nests originating on both sides of the Atlantic Ocean, including soil and wood feeders. Sampling locations encompassed tropical rainforest and savannah habitats. Our first aim was to better characterize the drivers of microbiome differences and to provide insights into the population structure and functions as they could be reflected in the metabolite and gene content.

### 4.1. Nest metabolomic composition

The metabolite untargeted approach stratified the samples according to feeding strategies, with a certain influence of the geographical location and genera (Figure 1). Separated PLS-DA models could differentiate between genera, geographical location, and feeding group (Supplementary Figures 1, 2). The 10 most decisive analytes were mainly alcohols, their derivates, and terpenic compounds.

One of the most important compounds for the separation of samples according to feeding habits was 2-butanol (Table 2A), which can originate from the decomposition of organic matter in the soil. Its methyl derivate, 2-methyl-butanol, was previously reported to have antifungal activity (Matsuura and Matsunaga, 2015). Another two important compounds for this separation are diacetyl (2,3-butanedione) and 2,3-pentanedione, potentially originating in higher amounts in wood feeders' nests because of fermentation (Yamaguchi et al., 1994). Other compounds important for separation and more abundant in wood feeders' nests were mostly terpenes.

Terpenes are a group of aromatic compounds that have been related to the mediation of information transmission (Gershenzon and Dudareva, 2007) or have been tested specifically as a toxic compound against termites (Seo et al., 2014). Among them, we established terpinolene as an analyte with decision power. Terpenes have been reported repeatedly in termite literature as a chemical component of the defensive secretions in *Nasutitermes* soldiers (Prestwich et al., 1981), not related to feeding habits as explained by its absence in workers. Besides, terpenes have been appointed as an antimicrobial substance, as confirmed by Mitaka et al. (2017). Terpinolene was first identified in the cephalic secretion from *Amitermes herbertensis* (Moore, 1968); and later in small quantities of a Nasutitermitinae, *Grallatotermes africanus* (Prestwich, 1979); in the defensive secretion from *Nasutitermes ephratae* soldiers (Valterová et al., 1986); soldier frontal glands in *Termitogeton planus* (Dolejšová et al., 2014); and significant amounts in *Constrictotermes cyphergaster* soldiers (De Mello et al., 2021). We found this compound mostly in the nests from *Nasutitermes*, with neglectable concentrations in others.

Our data indicated a lower level of naphthalene in comparison with published literature (Chen et al., 1998b; Wilcke et al., 2000). Overall, two genera concentrate the readings of naphthalene (*Nasutitermes* and *Cubitermes*), while the rest of the samples had a scattered representation. We did not record naphthalene in the nests from *Anoplotermes*. The type of sample preparation could have influenced, although a soil standard indicated correctness in our method. In some cases, the results could have been affected by the time elapsed from sampling to processing, a matter of a few days due to traveling and transportation. Nevertheless, we could still observe clear differences between samples at the volatile level that were further confirmed at the other omic approaches.

### 4.2. Nest microbial composition and structure

#### 4.2.1. Amplicon sequencing: Bacteria

The whole set of samples showed similar results to the overall untargeted metabolite profile without a clear stratification (Supplementary Figure 1). We observe an effect of phylogenetic relatedness and an influence of the geographical origin in the richness of the microbial communities associated with different nests. The highest values were those from breeds, potentially owed to an artificial input of strains from samples and colonies kept in similar laboratory conditions and proximity, and the potential input of human microbiome. The second highest level was that of MW samples. We hypothesize that the savannah conditions could pose a higher degree of stress over soil microbial communities, which could find a better shelter in these nests. Our β-diversity results show significant statistical differences between feeding Groups III and IV and between FG and MW.

At the ASVs-annotated PCA plot, samples with at least three representatives, we confirmed that the differences between microbial communities are determined strongly by the genera and diet (Figure 3A). While *Anoplotermes* and *Neocapritermes* belong to the same feeding group, they occupy distant locations in the plot, which provides the idea that genus is a stronger component than feeding strategy. The second part of the plot (Figure 3B) indicates that the geographical location is weaker when explaining the variability among nest bacterial communities based on 16S amplicon data. The statistical analysis through ANOSIM agrees with the previous analysis.

Our results agree with the published termite literature regarding the bacterial taxonomic composition of nests. *Actinomycetales* were reported as dominant in termite nests (Krishanti et al., 2018). These are Gram-positive bacteria capable of forming branching hyphae, related to nutrient recycling roles (Goodfellow and Williams, 2003), and have been used for the production of secondary metabolites, accounting for a very large fraction of all bioactive secondary metabolites in use in industry (Olano et al., 2008). This group has been also reported in the study by Chouvenc et al. (2018), where authors studied the origins of the acquisition of *Streptomyces*, the largest genus within *Actinobacteria* (El-Naggar, 2021), in *Coptotermes* nests. Authors have reported recruitment from surrounding soil. Similar results regarding *Streptomyces* were reported in the nests of *Reticulitermes flavipes* (Aguero et al., 2021). *Clostridiales* are Gram-positive anaerobic bacteria (Bowman, 2011) that can form resistant spores (Paredes-Sabja et al., 2011), described as thermophilic ethanologens capable of fermenting lignocellulosic biomass (Arora et al., 2019), including species that are lignocellulolytic (Auer et al., 2017). This order has been associated with the termite gut community of soil feeders (Ohkuma and Brune, 2010), and also *Reticulitermes santonensis*, through the presence of *Clostridia*-related clones within *Firmicutes* (Yang et al., 2005) and was appointed as one of the groups carrying out recycling of uric acid (Thong-On et al., 2012). In *Odontotermes yunnanensis*, Long et al. (2010) reported the presence of a wide representation of bacteria from the *Clostridium* genus in fungus comb structures. We found that this group was of the highest proportion in two of the soil feeders (*Anoplotermes* and *Cubitermes*) but also in nests from *Nasutitermes*. Regarding *Acidimicrobiales*, we have found no specific information in termite literature. *Solirubrobacterales* is an order with a few species described as Gram-positive mesophilic bacteria growing in a moderate range of temperatures (Whitman and Suzuki, 2015). Our results are in agreement with those described by Enagbonma et al. (2020), who report this group at termite mounds from *Coptotermes* species. Uncultured representatives of *Solirubrobacterales* have been associated with the degradation of lignin in soils (Wilhelm et al., 2018; Silva et al., 2021), also previously reported in sugarcane farm soils (Ogola et al., 2021).

*Spirochaetales* are Gram-negative that can comprise both anaerobic and strict aerobic bacteria (Smibert and Johnson, 1973). They have been reported in increased numbers when manure is applied to soil (Rieke et al., 2018). This group was also reported in *R. santonensis* gut (Yang et al., 2005) and gust from *Coptotermes gestroi* (Do et al., 2014) and have been appointed as active cellulose degraders (Xia et al., 2014), with abundance in wood-feeding termites (Tokuda et al., 2018; Hu et al., 2019). *Gemmatales* are Gram-negative chemoheterotrophic bacteria (Dedysh et al., 2020), which could act as an indicator of elevated phosphorus content in soil (Mason et al., 2021), and in a complex microbial community dedicated to the utilization of cellulose could be providing endocellulases and β-glucosidases activities (McDonald et al., 2019). Another group of bacteria notably described in the literature is that of *Rhizobiales*, Gram-negative bacteria belonging to the *Alphaproteobacterial* class, and with many members capable of fixing nitrogen (Beeckmans and Xie, 2015) and associated with many plants, lichens, and mosses (Erlacher et al., 2015). This order was also reported as enriched in termite nests of *Coptotermes testaceus, Heterotermes tenuis*, and *Nasutitermes octopilis* (Soukup et al., 2021). In our case, the proportion is limited.

Biomarkers analysis (LEfSe, Figure 4) indicated that certain genera were representative of specific samples. *Streptomyces* was associated with *Nasutitermes*, in agreement with Chouvenc et al. (2018) and Aguero et al. (2021), and could be indicative of a defensive role of this bacterial order in the termite nest. *Neocapritermes* with β-proteobacteria are described as characteristic cellulolytic bacteria in acidic forest soils in temperate areas (Štursová et al., 2012). Belonging to this class, the genera *Burkholderia* and *Collimonas* were characterized as capable of carrying out mineral weathering (Lepleux et al., 2012). Nest microbial communities from *Cubitermes* were associated with the genus *Rhodococcus*, comprising aerobic Gram-positive bacteria. Members from this genus have been associated with the degradation of nitroaromatic compounds (Subashchandrabose et al., 2018), but more importantly with lignin degradation activities (Chong et al., 2018). This observation is aligned with the fact that lignin degradation is not important in wood feeders (Ohkuma, 2003; Griffiths et al., 2013) and could be an indication that members of this genus carry this important task in the *Cubitermes-*associated communities. *Chitinophaga* has been described as a chitinolytic and ligninolytic genus (Funnicelli et al., 2021), besides *Spirochaeta*, where one of the species was reported to degrade complex plant polysaccharides and depolymerize lignin (Pandit et al., 2016). That observation agrees with the type of diet in *Anoplotermes*, which will find lignin-enriched sources in the soil, and by the construction of the nest, this type of bacteria can be enriched. Additionally, we could speculate that this bacteria is in charge of eliminating the growth of hyphae from fungi due to its ability to grow on fungal material (McKee et al., 2019). Perhaps, the role of this genus in the *Anoplotermes* nest could be related to the recycling of dead material from fungi.

#### 4.2.2. Amplicon sequencing: Fungi

The ACE index calculations informed of a lack of differences in the geographical origin and species. Differences exist according to diet and phylogenetic relatedness, which can drive a differential richness in the nest fungal communities.

The PCA plot built in R (Figure 5) indicated a stronger effect of genera and feeding strategy. We believe that genera were even stronger in sample stratification than the type of diet because even two genera in the same feeding group are clearly separated (*Anoplotermes* and *Neocapritermes*). Finally, while it existed, the geographical influence was weaker than the other two. These results are in overall agreement with our observations of the bacterial communities. In keeping with these results, ANOSIM showed a stronger influence of diet and genus over geographical origin.

#### 4.2.3. Shotgun metagenomic sequencing

We have used different ways of presenting these data due to the massive amount of information that could be extracted. Feeding group and genera stratify samples with limited influence from the geographical location (Figure 6). Despite small variability between nests from species belonging to the same genus, even communities from soil-feeding termite nests show closer genomic and taxonomic composition at both sides of the Atlantic, as revealed by the NMDS analysis that takes into account the full metagenome.

At the phylum level (Figure 7A), we observe a comparable situation, where all *Nasutitermes* have a closer profile among themselves than those of *Cubitermes* or *Anoplotermes*. There are still differences between samples within the same genera (except for *Nasutitermes*). One of the samples from *Anoplotermes banksi* shows large differences with the other two samples from this species, probably owing to the worst state of that sample when arriving in our laboratory. The three samples from *Nasutitermes lujae* are very stable in terms of taxonomic representation. We can conclude that the taxonomic composition of the nests reflects the genera (where it seems to be stable) and especially eating habits. Taxonomic metabarcoding through 16S amplicon sequencing shows similar trends with the metagenomic bacterial annotations, with a notable presence of *Actinobacteria*. However, that is not the case for *Proteobacteria*. We found that the most abundant order in many of the sequenced soil feeders was *Streptosporangiales*, whereas *Nasutitermes* belong to the class *Actinomycetia*, either to genera *Gryllotalpicola* in three cases or *Mycobacterium* in the last one. Differences in the results of both sequencing approaches have been previously addressed in the literature (Rausch et al., 2019).

Metagenomics can provide very interesting insights into the microbial community by dissecting the functional potential. Given a certain function, we could observe if at that level exists a differential composition in terms of taxa. We carried out a subset of functions (Puente-Sánchez et al., 2020) related to “phenylalanine, tyrosine, and tryptophan biosynthesis.” These three aromatic α-amino acids are involved in the metabolism of secondary metabolites (Parthasarathy et al., 2018). We found apparent differences according to the feeding strategies (Figure 7B). Once more, both soil-feeding genera resemble each other, while the *Nasutitermes* keep a closer profile among them. This indicates that the differences are not only at the metabolomic level but also at the overall taxonomic structure of the community (both amplicon and metagenomic sequencing data). The differences go beyond this, and certain functions are carried out by different microorganisms. While nests from soil-feeding termites display similar profiles on both sides of the Atlantic, those from wood and soil feeding are more dissimilar. Considering that, the geographical component seems to have a weaker contribution than the feeding habits in driving the functional differences among communities. There seems to be a small reflection of a component that relates communities from the African environment, as we observe a group of unclassified *Actinobacteria* with a similar proportion between *Nasutitermes* and *Cubitermes*.

Our explanation here is that the feeding habits of termites and possibly some termite-active antimicrobial strategies are creating a set of conditions that allow the growth of certain types of genera, which will carry the required set of community functions, in this case, the metabolism of aromatic amino acids. We mentioned the termite active strategies because our study included terpene as a selective metabolite, which has antimicrobial properties and is secreted by *Nasutitermes*, as both are documented in the literature. The defensive or protective component of feces was greatly reviewed by Cole et al. (2021). In our study, we inferred that the combination of active strategies and passive constraints imposed by the feeding strategy created a specific growth medium for selected microorganisms. We could establish parallelism with microbiology work in the laboratory and state that termites with different feeding habits have formulated a growth medium for their allied microbiome to thrive. Several iterations of this process through trial and error in evolutionary time have probably fine-tuned these strategies, where the most successful in ensuring the nest and colony survival have been fixed. To such an extent that microbial communities associated with two different genera of soil feeders resemble in structure on both sides of the Atlantic Ocean, where the different flora composition imposes a different soil community (Lima-Perim et al., 2016). However, despite these stated differences even at short distances belonging to two clearly distinct biotopes, the differences between the *Cubitermes* from CM and MW are minor. Thus, this is one more observation that helps us to associate a lower discrimination power to the geographical location. The recruitment of external or surrounding strains reported in the literature can be different in terms of specific strains, but they will be fundamentally equivalent in the functions that they carry out. Soil feeders “will recruit” microorganisms that are performing or carrying the same genes. We want to highlight that “recruit” may not be an active termite program, but a consequence of the complex crosstalk between active antimicrobial strategies, and the effect of diet and phylogenetic relatedness.

Among the communities with a higher presence in *Nasutitermes*, the genus *Gryllotalpicola* has been found in larval galleries from the pine beetle *Monochamus alternatus* collected in the Chinese Jiangxi province (Chen et al., 2020) and was previously isolated from the guts of the wood-feeding *Reticulitermes chinensis* Snyder, sampled in Wuhan, China (Fang et al., 2015). The interesting connection is that members from this genus have been found in insects with wood-feeding habits at distant locations. *Streptosporangiales* is an order of bacteria that can obtain carbon from green waste (Cai et al., 2018), especially at the middle composting stage (Li et al., 2021). They can secrete cellulases and hemicellulases for lignocellulosic degradation, besides being producers of antibiotics that suppress pathogens (Gomes et al., 2017; Waglechner et al., 2019). These organisms are highly abundant in soil (McCarthy and Williams, 1992). Thus, since soil-feeding genera use soil as their substrate, *Streptosporangiales* may originate in their surrounding environment and be enriched due to the substrate type and nutrients present. They could provide a cleaner environment at the termite nest due to the production of antimicrobial compounds. This strategy, which we cannot ascertain if it is an active strategy or one derived from the termite activity, repeats itself on both sides of the Atlantic Ocean. The genus *Microlunatus* (*Actinobacteria*) has been previously reported in *Mycocepurus smithii* ants (Kellner et al., 2015), a fungus-farming ant reported to acquire its microbiome from the surrounding environment. Bacteria from this genus have been found in deep-sea sediments (Jroundi et al., 2020) to rhizospheric soil (Wang et al., 2008), and it has been indicated that they have the potential for pollution management (Zhang et al., 2017), especially for capabilities of phosphorus accumulation (Zhong et al., 2018). *Microbacteriaceae* is a family of Gram-positive bacteria with members that have been reported among the dominant cellulase-producing strains isolated from yellow stem borers (*Scirpophaga incertulas*; Bashir et al., 2013), but in much less proportion in the guts of termites at the same study identified as *Odontotermes hainanensis*, which were feeding on decomposed trees and leaf litter.

The LEFSE analysis on the ORFs annotated at PFAM has found that each of the genera has a specific signature (Figure 8), which was also a conclusion that we could get after the quick interrogation of PFAM annotation (Supplementary Figures 4, 5). There are differences when we seek terms that are related to key aspects of the microbial lifestyle (e.g., cellulose-annotated genes). But are these apparent differences in specific functions translated into differential content of certain genes beyond a LEFSE analysis or our quick approach? Has the microbial community gained or is being enriched in certain functions? To answer these questions, we applied a transcriptomics statistical test for finding differentially expressed genes (DGEs); in our case, we adopted the term DPGs. Testing for significant statistical differences will illustrate if a certain function is highly abundant in a nest microbial community when compared with another. We must observe that in a metagenomics context, we will not get an idea of “active use” but more of “potential use.” While not all present genes will be expressed, we can conclude that a content increase in certain functions is relevant and not random. That is the rationale behind using qPCRs to quantify the differences in gene content due to pharmaceutical pollution among different sites (González-Plaza et al., 2019; Milaković et al., 2019).

While TPM is a normalization method generally used in RNAseq experiments and its data treatment, according to Puente-Sánchez et al. (2020), it can be very useful in metagenomic experiments. In that regard, we found it to be a very important value that allows for sample comparison and general overviews (Figure 6). However, because we understand the limitations of TPM use (Zhao et al., 2020), we have carried out additional normalizations for comparison purposes using procedures that use raw count values and are normalized differently (González Plaza, (in preparation).

Comparison based on the normalized count values for the PFAM-annotated ORFs clearly separates *Nasutitermes* from the soil feeders (Figure 9), again a division by the termite diet and phylogenetic relatedness. We found that the differences between the microbial communities in wood-feeding termites' nests and those in soil-feeding termites' nests are much stronger. We carried out a simple analysis, extracting terms related to the different metabolisms of the communities (e.g., cellulose; Supplementary Figures 4, 5), where we observed genes such as cellulose synthases that play a strong role in *Nasutitermes*.

This led us to carry out a differential gene content analysis with the results obtained in specialized databases and observed a similarity among soil feeders and great differences to the wood feeder genus. The differences in the use of CAZy-annotated genes were small and absent between both soil feeders. We must note that this could be a resemblance to the smaller number of genes that we used for this analysis due to computing limitations. If proportions are kept regarding what we observe in the other comparisons, a higher number of DPGs between *Nasutitermes* and each of the soil feeders, it is logical to think that we did not have enough number of “events” for statistical differences to appear. We found interesting differences, as three of the most differentially present genes in the wood-feeder nest belong to the glycosyl transferase family (GT 2, 4, and 51), previously reported in woodchip communities but not related to the cellulose hydrolysis (Nnadozie et al., 2017).

The rationale for this additional genetic prospecting is that these databases can reflect different aspects of the microbial communities, which can be manifold and related to metabolism as a cornerstone (Schmidt et al., 2019). Genes annotated in AromaDeg could serve for protection purposes when organic antimicrobial compounds such as terpenes are used, but they could also be part of a metabolism that could drive social microbial interactions. The degradation of aromatic hydrocarbon compounds was shown in the guts of wood-feeding termites (Ke et al., 2011), while in our study, the microbial communities could be utilizing lignin-related aromatic compounds. The differences observed between wood and soil feeders (in their nests) are owed to the different inputs of compounds due to the diet, as soil feeders have a great input of polyaromatic compounds (Ohkuma, 2003). Antibiotic resistance can be used among members of the community to defend themselves against antibiotics, either from those produced by them or from competing organisms (Scherlach and Hertweck, 2020). Additionally, at the usual concentrations of antibiotics found in natural communities, these genes can serve for communication purposes rather than survival (Romero et al., 2011). Besides, we found it interesting to query the CARD database because the antibiotic resistance genes harbored in wildlife-associated microbial communities have not been properly addressed (Dolejska and Literak, 2019). The low number of described genes at BactiBase or AnhyDeg indicates the potential of these termite nest microbial communities for being a source of novel genes that can be used in fighting antimicrobial resistance (bacteriocins). MacB was one of the most dominant genes in *Nasutitermes*, a gene reported in several cultivation ecosystems (Fan et al., 2021).

Hu et al. (2019) suggested the linkage between diet-microbiome in termite guts, a widespread observation in other animal models (Colman et al., 2012; David et al., 2013; Baker et al., 2014; Flint et al., 2015; Otani et al., 2019; Leite-Mondin et al., 2021). However, through three omics approaches, we state that these differences go beyond the gut. Diet shapes the nest community structure because of the building strategy, without forgetting that there is a contribution of phylogenetic relatedness fine-tuned by the geographical location.

## 5. Conclusion

We found differences in the overall metabolite profile indicating a stronger effect of the feeding habits and phylogenetic relatedness than the geographical location, where some selective metabolites may have an antimicrobial role. Naphthalene appeared in nests from *Cubitermes* and *Nasutitermes*.

The bacterial and fungal taxonomical profiles point toward a stratification mainly owed to diet and phylogenetic relatedness, which is potentially explained since nest material is a product of termite feces and surrounding soil. Even the differences between soil-feeding termites' nests from both African sites were minor.

Metagenomic sequencing supports the above results, where the major drives for genetic functional differences are diet (greater effect over fungi) and phylogenetic relatedness (greater effect over bacteria). The genetic differences between both soil-feeding termites were of a more limited entity than those between the wood-feeding ones. Our mining approaches with specialized databases show a vast genetic richness of functions and genes for biotechnological applications. This is only one example of the virtually unlimited genetic diversity that tropical rainforests harbor and should draw attention to the necessity of preserving this resource that could be applied in fields such as the bioremediation of organic compounds or the synthesis of biofuels.

Many studies have indicated that termites recruit microbial members from the surrounding environment. Our results also indicate that the microbiome genetic functions in soil-feeding termites are similar across the Atlantic, even in savannah and rainforest regions. What ultimately fixes the functional genomic content is diet, the nutrients that are available, where diet is a product of evolutionary history.

Translated into our world, controlling the nutrients that microbial communities receive or are exposed to leads to dramatic changes in the composition and, more importantly, the functional genetic content. These results can inspire methodologies to drive the composition and functions of microbial communities and limit the spread of pathogens.

Further studies considering the different microbial niches (gut, environment, food substrate, and nest) are necessary to understand the relationship between the microbial communities under the influence of termites and those in the surrounding environment.

## Data availability statement

The datasets presented in this study can be found in online repositories. The names of the repository/repositories and accession number(s) can be found at: https://www.ebi.ac.uk/ena, PRJEB55779.

## Ethics statement

Sampling was carried out in accordance with the Nagoya guidelines for biodiversity sampling (Convention on Biological Diversity, 2010), with the required research permits to Jan Šobotník and other members of FFWS, and in accordance with the ethical behavior toward local communities.

## Author contributions

JG and JH designed the study. JG participated in sample collection, designed the sampling strategy, isolated DNA and COII-identified many specimens used in this study, processed nest material to powder, carried out all experiments regarding nest DNA isolation, sequencing data processing, analysis, and wrote the main body of the manuscript. JH performed the metabolite profiling setup, experiments, data processing, writing of the corresponding metabolite sections in the manuscript, and participated in article writing and data interpretation. All authors contributed to the article and approved the submitted version.
