# Supplementary figures and images for: The tropical cookbook: Termite diet and phylogenetics—Over geographical origin—Drive the microbiome and functional genetic structure of nests
Source: Front Microbiol. 2023 Mar 14;14:1089525. doi: 10.3389/fmicb.2023.1089525 (PMC10043212; doi:10.3389/fmicb.2023.1089525)

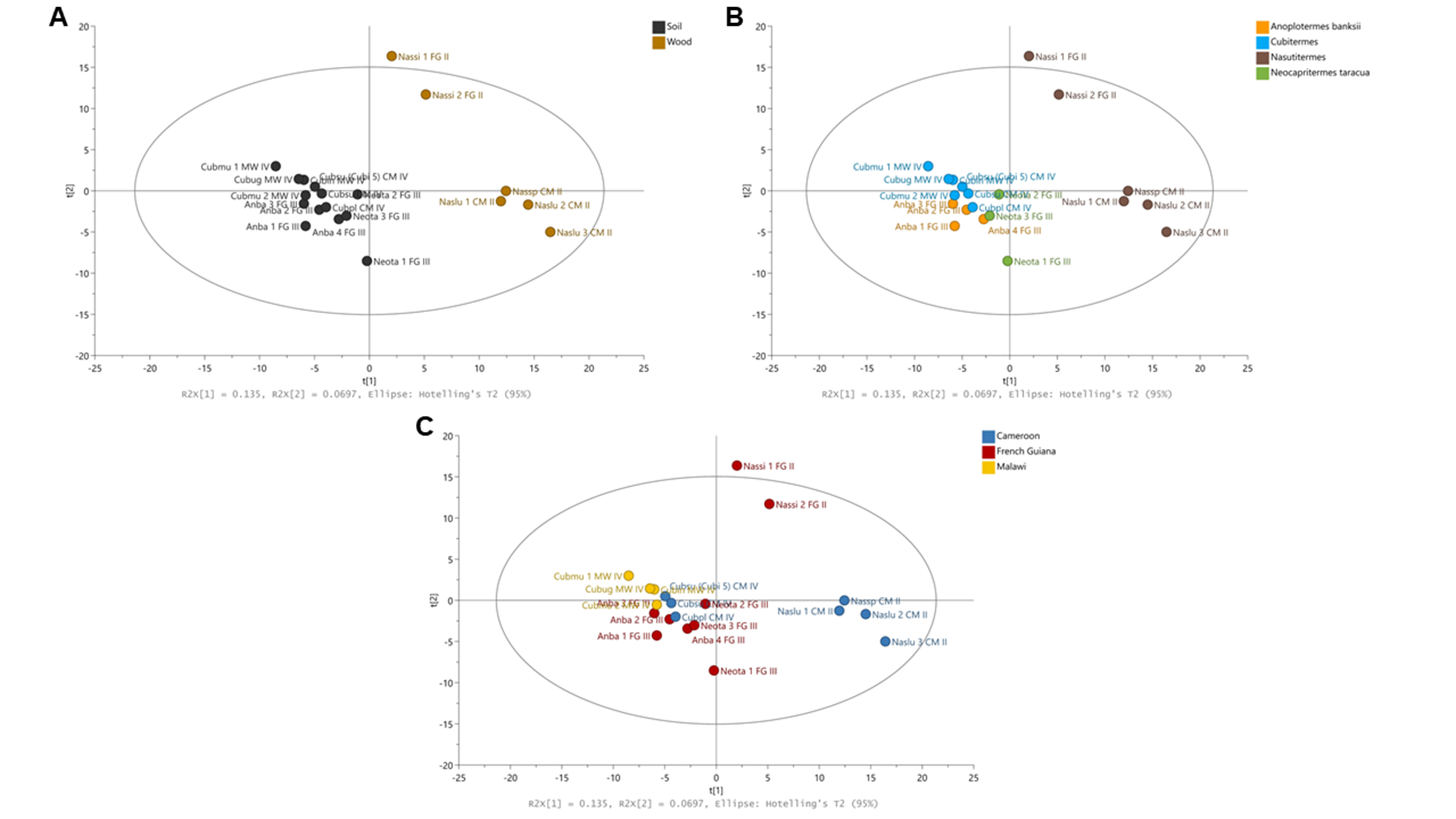

Supplement: Supplementary file 13 [file Image_1.TIF]

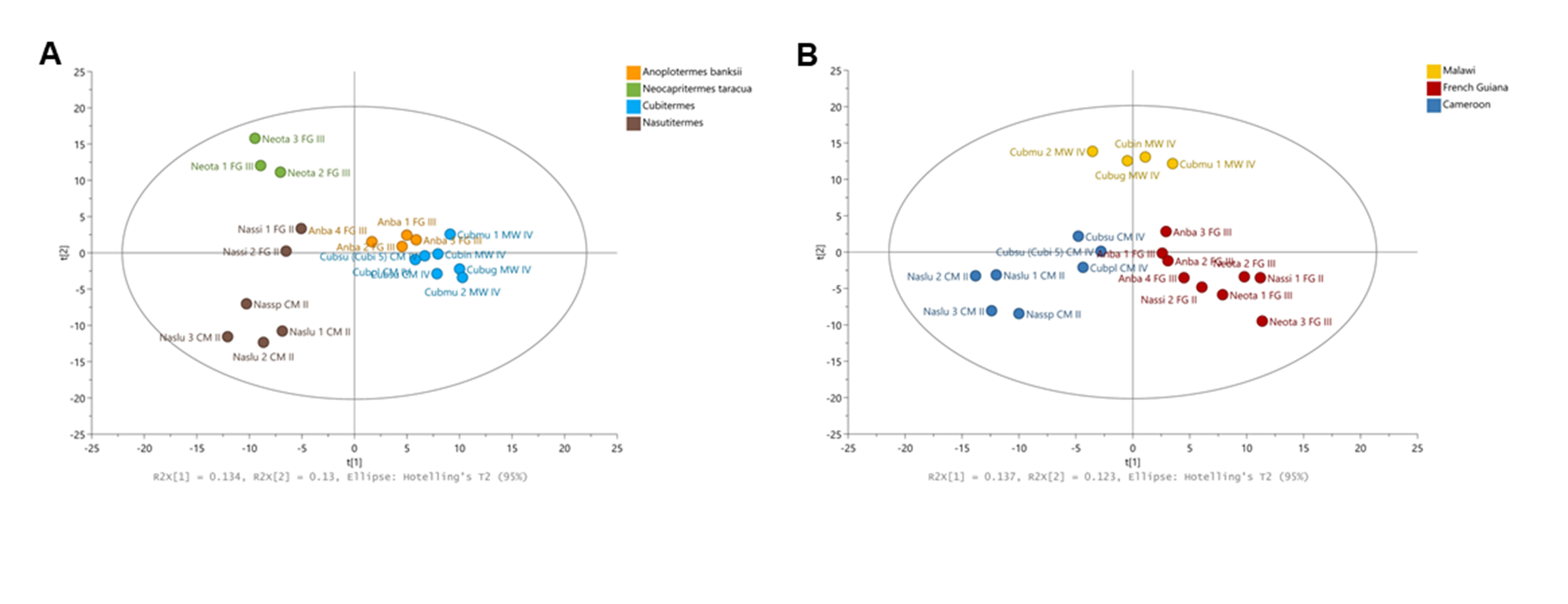

Supplement: Supplementary file 14 [file Image_2.TIF]

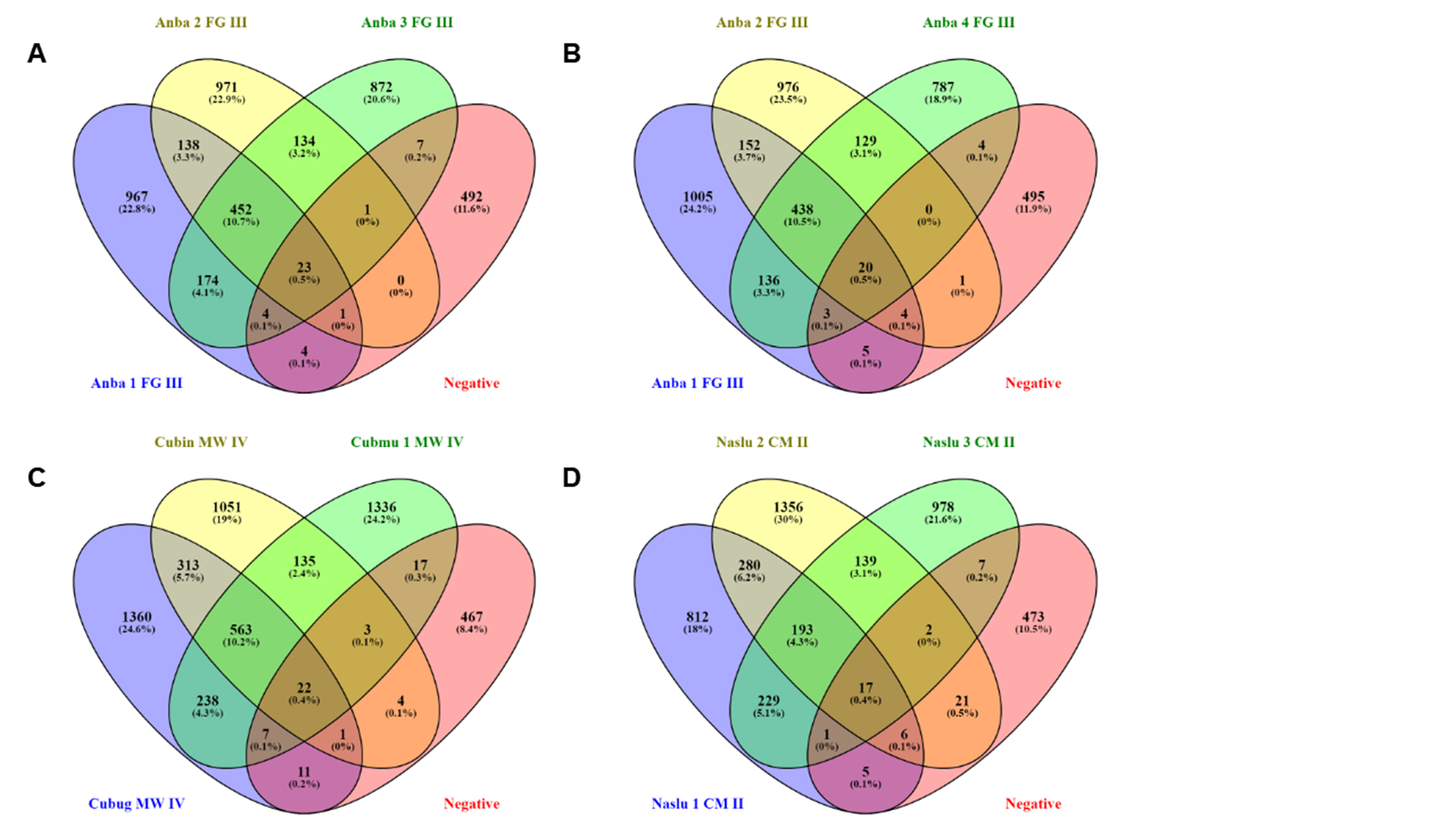

Supplement: Supplementary file 15 [file Image_3.TIF]

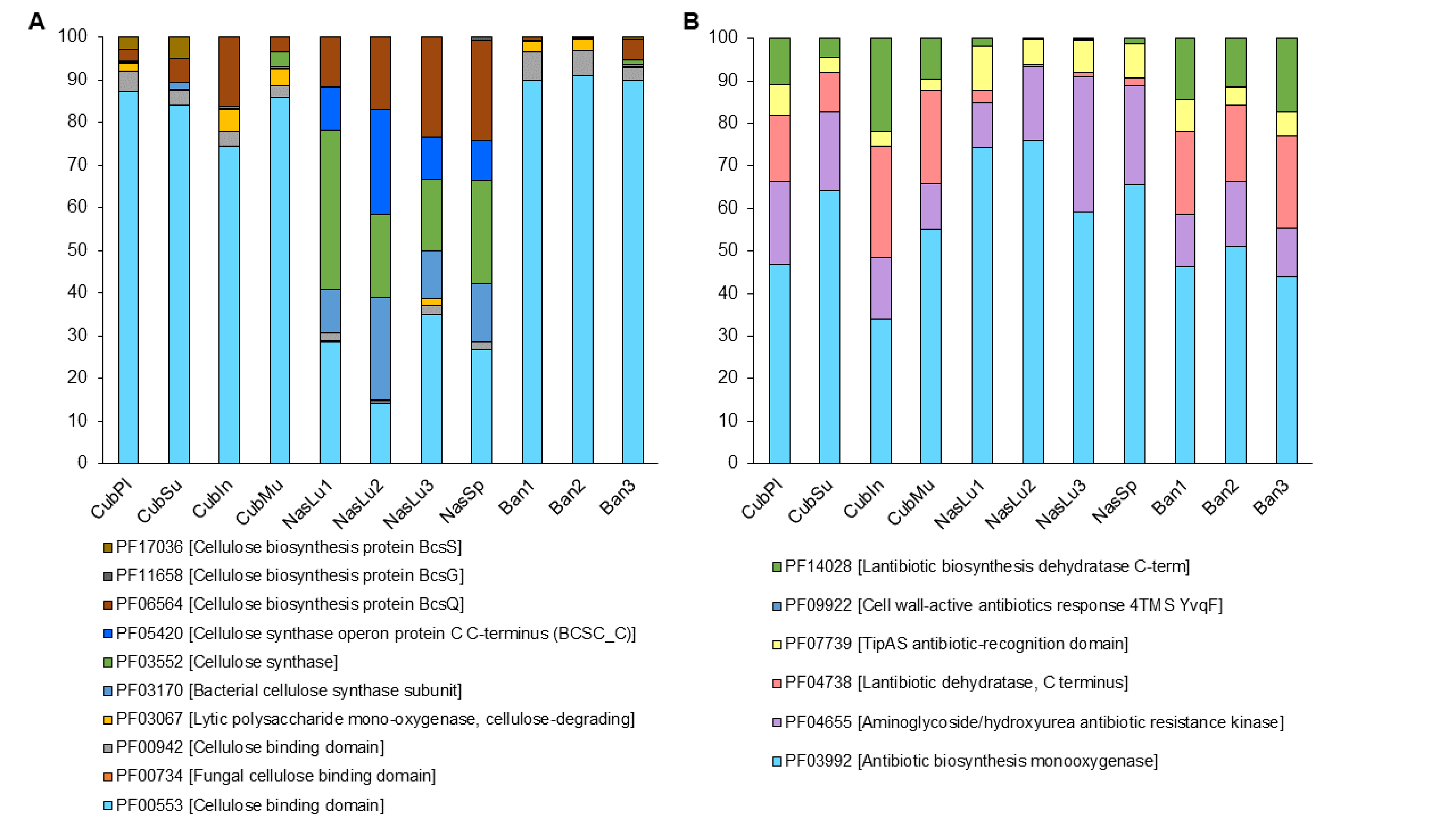

Supplement: Supplementary file 16 [file Image_4.PNG]

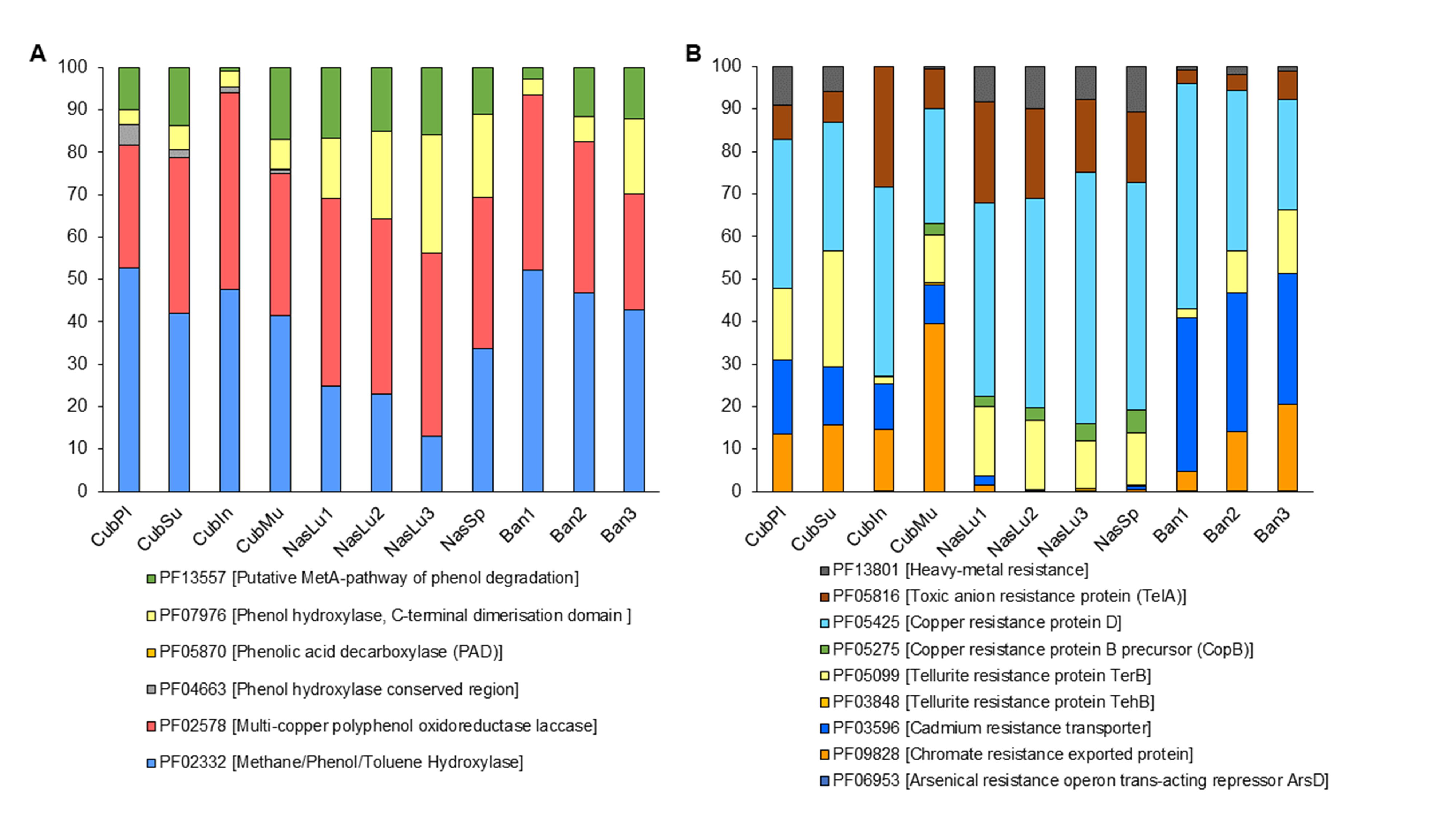

Supplement: Supplementary file 17 [file Image_5.TIFF]

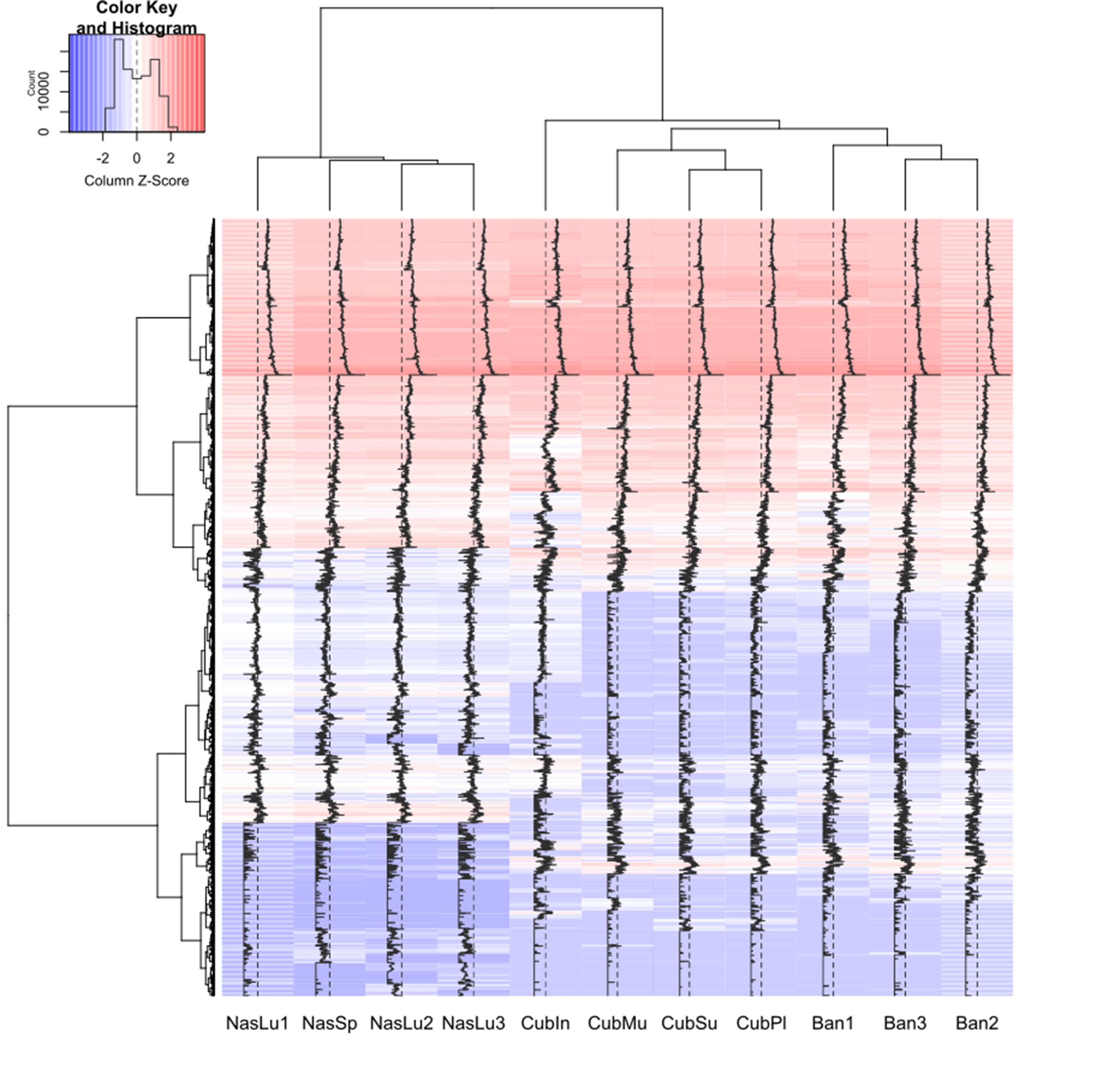

Supplement: Supplementary file 18 [file Image_6.TIFF]

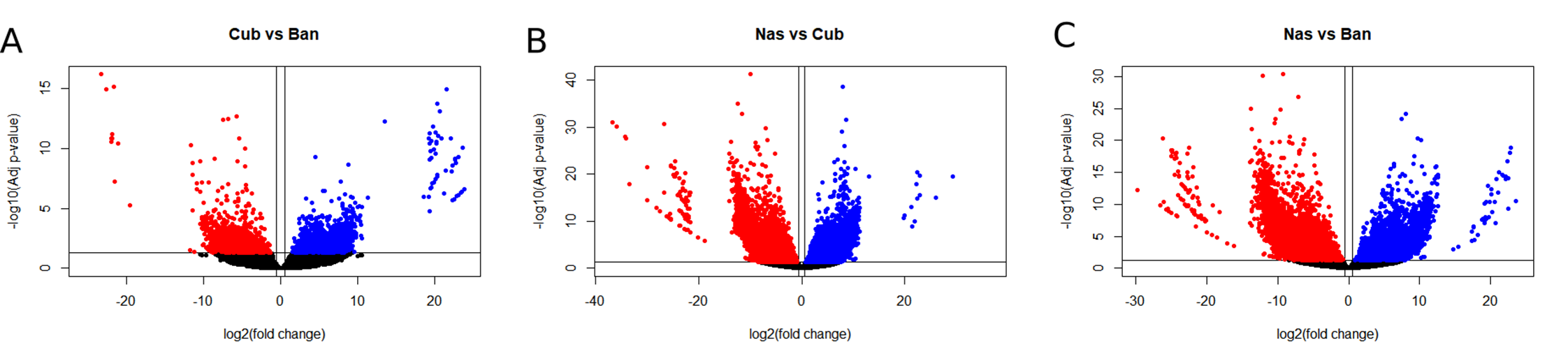

Supplement: Supplementary file 19 [file Image_7.TIFF]
