# Supplementary material for: The tropical cookbook: Termite diet and phylogenetics—Over geographical origin—Drive the microbiome and functional genetic structure of nests
Source: Front Microbiol. 2023 Mar 14;14:1089525. doi: 10.3389/fmicb.2023.1089525 (PMC10043212; doi:10.3389/fmicb.2023.1089525)
Supplement: Supplementary file 20 [file Table_1.DOCX]

**Table S1.** Statistics on metagenomic sequencing. Raw reads: total amount of reads of raw data, each four lines taken as one unit. For paired-end sequencing, it equals the amount of read1 and read2, otherwise it equals the amount of read1 for single-end sequencing. Seq.: sequencing; Geo.: Geographical. Raw data: (Raw reads) * (sequence length), calculating in G. For paired-end sequencing like PE150, sequencing length equals 150, otherwise it equals 50 for sequencing like SE50. Effective: (Clean reads/Raw reads) * 100. Error: base error rate. Q20, Q30: (Base count of Phred value > 20 or 30) / (Total base count). GC: (G & C base count) / (Total base count)

| **Geo. location** | **Species** | **Code** | **Sample seq. code** | **Data treatment code** | **Raw reads** | **Effective (%)** | **Error (%)** | **Q20 (%)** | **Q30 (%)** | **GC (%)** |
| --- | --- | --- | --- | --- | --- | --- | --- | --- | --- | --- |
| FG | *Anoplotermes banksii* | Anba 2 FG III | Ban2 | Ban1 | 53,288,002 | 99.67 | 0.02 | 98.3 | 95.44 | 66 |
|  |  | Anba 3 FG III | Ban3 | Ban2 | 55,905,810 | 99.7 | 0.02 | 98.4 | 95.59 | 65.8 |
|  |  | Anba 4 FG III | Ban4 | Ban3 | 66,039,800 | 99.59 | 0.03 | 97.6 | 93.83 | 68.3 |
| CM | *Nasutitermes lujae* | Naslu 1 CM II | Nal1 | NasLu1 | 63,479,926 | 99.68 | 0.02 | 98 | 94.97 | 68.3 |
|  |  | Naslu 2 CM II | Nal2 | Naslu2 | 53,196,396 | 99.67 | 0.02 | 98.1 | 95.17 | 68.3 |
|  |  | Naslu 3 CM II | Nal3 | Naslu3 | 63,131,466 | 99.66 | 0.02 | 98.1 | 95.14 | 67.5 |
|  | *Nasutitermes sp.* | Nassp CM II | NasSp | NasSp | 56,112,754 | 99.7 | 0.02 | 98.2 | 95.34 | 68.1 |
|  | *Cubitermes planifrons* | Cubpl CM IV | CubPL | CubPl | 49,692,682 | 99.74 | 0.02 | 98.2 | 95.35 | 67.1 |
|  | *Cubitermes sulcifrons* | Cubsu CM IV | CubSu | CubSu | 55,052,860 | 99.71 | 0.02 | 98.2 | 95.25 | 67 |
| MW | *Cubitermes muneris* | Cubmu 1 MW IV | MLW3 | CubMu | 56,193,314 | 99.75 | 0.02 | 98.2 | 95.24 | 66.4 |
|  | *Cubitermes inclitus* | Cubin MW IV | MLW2 | CubIn | 53,443,938 | 99.7 | 0.02 | 98.2 | 95.34 | 69.1 |
|  |  |  | Total |  | 625,536,948 |  |  |  |  |  |

**Table S2.** Statistics on metagenomic sequencing. Abund.: abundant; Superk: superkingdom; Actinob.: Actinobacteria; Actinom.: Actinomycetia; Streptosp.: Streptosporangiales; Hyphom.: Hyphomicrobiales; Microc.: Micrococcales.; Microb.: Microbacteriaceae.; Gryllot.: Gryllotalpicola.; Dormib.: Dormibacteraeota; Alphaprot.: Alphaproteobacteria. Note: “Data treatment code” (Table 4) has been used to name samples, for simplicity purposes.

| **Most abund.taxa** | **Ban1** | **Ban2** | **Ban3** | **CubIn** | **CubMu** | **CubPl** | **CubSu** | **NasLu1** | **NasLu2** | **NasLu3** | **NasSp** |
| --- | --- | --- | --- | --- | --- | --- | --- | --- | --- | --- | --- |
| Superk. | Bacteria | Bacteria | Bacteria | Bacteria | Bacteria | Bacteria | Bacteria | Bacteria | Bacteria | Bacteria | Bacteria |
| phylum | Actinob. | Actinob. | Actinob. | Actinob. | Actinob. | Actinob. | Actinob. | Actinob. | Actinob. | Actinob. | Actinob. |
| class | Actinom. | Actinom. | Actinom. | Actinom. | Actinom. | Actinom. | Actinom. | Actinom. | Actinom. | Actinom. | Actinom. |
| order | Streptosp. | Streptosp. | Streptosp. | Streptosp. | Hyphom. | Streptosp. | Streptosp. | Hyphom. | Hyphom. | Microc. | Microc. |
| family | Pseudonocardiaceae | Bradyrhizobiaceae | Mycobacteriaceae | Nocardiopsaceae | Bradyrhizobiaceae | Nocardiopsaceae | Mycobacteriaceae | Bradyrhizobiaceae | Microb. | Microb. | Microb. |
| genus | Pseudonocardia | Mycobacterium | Mycobacterium | Microlunatus | Bradyrhizobium | Mycobacterium | Mycobacterium | Mycobacterium | Gryllot. | Gryllot. | Gryllot. |
| species | Actinob. bacterium | Bradyrhizobiaceae bacterium | Alphaprot. bacterium | Candidatus Dormib. bacterium | Alphaprot. bacterium | Actinob. bacterium | Actinob. bacterium | Acidimicrobiaceae bacterium | Gryllot. protaetiae | Gryllot. protaetiae | Gryllot. protaetiae |
|  |  |  |  |  |  |  |  |  |  |  |  |

**Table S3.** Statistics on ORFs after SqueezeMeta pipeline.

|  | **Whole Assembly** | **Ban1** | **Ban2** | **Ban3** | **CubIn** | **CubMu** | **CubPl** | **CubSu** | **NasLu1** | **NasLu2** | **NasLu3** | **NasSp** |
| --- | --- | --- | --- | --- | --- | --- | --- | --- | --- | --- | --- | --- |
| **Number of ORFs** | 8,127,615 | 1,397,157 | 2,467,662 | 2,392,339 | 1,577,504 | 2,470,918 | 2,701,353 | 2,768,071 | 1,107,237 | 1,044,972 | 1,167,809 | 1,152,944 |
| **Number of rRNAs** | 2,390 | 1,024 | 1,276 | 1,415 | 1,221 | 1,447 | 1,339 | 1,380 | 995 | 1,039 | 1,223 | 1,257 |
| **Number of tRNAs/tmRNAs** | 27,874 | 4,555 | 6,898 | 6,420 | 4,890 | 6,658 | 8,385 | 8,613 | 4,384 | 4,378 | 4,941 | 4,643 |
| **ORFs by Aragorn** | 27,874 | 4,555 | 6,898 | 6,420 | 4,890 | 6,658 | 8,385 | 8,613 | 4,384 | 4,378 | 4,941 | 4,643 |
| **ORFs by Prodigal** | 8,097,351 | 1,391,578 | 2,459,488 | 2,384,504 | 1,571,393 | 2,462,813 | 2,691,629 | 2,758,078 | 1,101,858 | 1,039,555 | 1,161,645 | 1,147,044 |
| **ORFs by barrnap** | 2,390 | 1,024 | 1,276 | 1,415 | 1,221 | 1,447 | 1,339 | 1,380 | 995 | 1,039 | 1,223 | 1,257 |
| **Orphans (no hits)** | 1,343,453 | 148,874 | 277,464 | 255,731 | 172,699 | 267,778 | 287,824 | 304,858 | 193,024 | 170,776 | 187,135 | 190,547 |
| **No tax assigned (with hits)** | 18,951 | 2,078 | 4,110 | 3,915 | 2,625 | 4,320 | 4,687 | 4,828 | 3,372 | 2,910 | 3,849 | 3,642 |
| **KEGG annotations** | 4,095,584 | 801,355 | 1,412,923 | 1,388,974 | 899,823 | 1,423,940 | 1,552,121 | 1,588,244 | 597,678 | 580,592 | 648,183 | 639,147 |
| **COG annotations** | 5,811,435 | 1,111,402 | 1,946,729 | 1,902,975 | 1,237,057 | 1,941,438 | 2,133,173 | 2,173,849 | 801,962 | 768,568 | 862,097 | 848,836 |
| **PFAM annotations** | 2,844,947 | 646,229 | 1,072,204 | 1,029,204 | 697,782 | 1,045,021 | 1,161,233 | 1,169,171 | 455,815 | 440,914 | 488,195 | 483,351 |

**Table S4.** Statistics on bins after SqueezeMeta pipeline. Qual.: quality; contam.: contamination.

| **Number of bins** | 47 |
| --- | --- |
| **Complete ≥ 50%** | 33 |
| **Complete ≥ 75%** | 16 |
| **Complete ≥ 90%** | 2 |
| **Contamination < 10%** | 22 |
| **Contamination ≥ 50%** | 3 |
| **Congruent bins** | 7 |
| **Disparity >0** | 40 |
| **Disparity ≥ 0.25** | 21 |
| **Hi-qual. bins (>90% complete, <10% contam.)** | 1 |
| **Good-qual. bins (>75% complete, <10% contam.)** | 3 |

**Table S5.** LEfSe biomarker categrories for the three genera included in the metagenomic sequencing.

| **Term** | **Genus** | **LDA Score Log10** |
| --- | --- | --- |
| PF00196 Bacterial regulatory proteins luxR family | *Anoplotermes* | 2.836470466 |
| PF13424 Tetratricopeptide repeat | *Anoplotermes* | 2.590311713 |
| PF03704 Bacterial transcriptional activator domain | *Anoplotermes* | 2.445492463 |
| PF13191 AAA ATPase domain | *Anoplotermes* | 2.442521088 |
| PF07719 Tetratricopeptide repeat | *Anoplotermes* | 2.411987663 |
| PF04392 ABC transporter substrate binding protein | *Anoplotermes* | 2.396870569 |
| PF00515 Tetratricopeptide repeat | *Anoplotermes* | 2.365780361 |
| PF13374 Tetratricopeptide repeat | *Anoplotermes* | 2.32394555 |
| PF07228 Stage II sporulation protein SpoIIE | *Anoplotermes* | 2.287137796 |
| PF00553 Cellulose-binding domain | *Anoplotermes* | 2.271918485 |
| PF13176 Tetratricopeptide repeat | *Anoplotermes* | 2.18787753 |
| PF14200 Ricin type beta trefoil lectin domain like | *Anoplotermes* | 2.172000085 |
| PF00652 Ricin type beta trefoil lectin domain | *Anoplotermes* | 2.146611872 |
| PF13185 GAF domain | *Anoplotermes* | 2.134791179 |
| PF00400 WD domain G beta repeat | *Anoplotermes* | 2.100263122 |
| PF00990 Diguanylate cyclase GGDEF domain | *Anoplotermes* | 2.093703518 |
| PF00211 Adenylate and Guanylate cyclase catalytic domain | *Anoplotermes* | 2.090632177 |
| PF13683 Integrase core domain | *Anoplotermes* | 2.084520819 |
| PF00872 Transposase Mutator family | *Cubitermes* | 2.230145835 |
| PF00884 Sulfatase | *Cubitermes* | 2.110764005 |
| PF00296 Luciferase-like monooxygenase | *Nasutitermes* | 2.86094335 |
| PF02515 CoA transferase family III | *Nasutitermes* | 2.627791182 |
| PF00873 AcrB/AcrD/AcrF family | *Nasutitermes* | 2.599203891 |
| PF00171 Aldehyde dehydrogenase family | *Nasutitermes* | 2.547507072 |
| PF04185 Phosphoesterase family | *Nasutitermes* | 2.499718767 |
| PF00126 Bacterial regulatory helix turn helix protein lysR family | *Nasutitermes* | 2.485956307 |
| PF12833 Helix turn helix domain | *Nasutitermes* | 2.474596527 |
| PF03466 LysR substrate-binding domain | *Nasutitermes* | 2.452533989 |
| PF13458 Periplasmic-binding protein | *Nasutitermes* | 2.44486032 |
| PF03972 MmgE PrpD family | *Nasutitermes* | 2.362281876 |
| PF13181 Tetratricopeptide repeat | *Nasutitermes* | 2.353027845 |
| PF00593 TonB dependent receptor | *Nasutitermes* | 2.343076365 |
| PF05685 Putative restriction endonuclease | *Nasutitermes* | 2.317998308 |
| PF01408 Oxidoreductase family NAD-binding Rossmann fold | *Nasutitermes* | 2.314995708 |
| PF13432 Tetratricopeptide repeat | *Nasutitermes* | 2.309689871 |
| PF19305 | *Nasutitermes* | 2.29442485 |
| PF13431 Tetratricopeptide repeat | *Nasutitermes* | 2.283032563 |
| PF01391 Collagen triple helix repeat 20 copies | *Nasutitermes* | 2.2539895 |
| PF02826 D isomer specific2 hydroxyacid dehydrogenase NAD-binding domain | *Nasutitermes* | 2.247235918 |
| PF13428 Tetratricopeptide repeat | *Nasutitermes* | 2.203654173 |
| PF13414 TPR repeat | *Nasutitermes* | 2.20286346 |
| PF09084 NMT1 THI5-like | *Nasutitermes* | 2.196745468 |
| PF00664 ABC transporter transmembrane region | *Nasutitermes* | 2.177136256 |
| PF07715 TonB dependent Receptor Plug Domain | *Nasutitermes* | 2.16487022 |
| PF13433 Periplasmic-binding protein domain | *Nasutitermes* | 2.148044669 |
| PF13371 Tetratricopeptide repeat | *Nasutitermes* | 2.102367924 |
| PF02661 Fic DOC family | *Nasutitermes* | 2.091561869 |
| PF01436 NHL repeat | *Nasutitermes* | 2.082221658 |
| PF04261 Dyp-type peroxidase family | *Nasutitermes* | 2.074442421 |
| PF02321 Outer membrane efflux protein | *Nasutitermes* | 2.072494344 |
| PF12849 PBP superfamily domain | *Nasutitermes* | 2.071868327 |
| PF14559 Tetratricopeptide repeat | *Nasutitermes* | 2.060797543 |
| PF07804 HipA likeC terminal domain | *Nasutitermes* | 2.048652465 |
| PF04255 Protein of unknown function DUF433 | *Nasutitermes* | 2.02745921 |
| PF01011 PQQ enzyme repeat | *Nasutitermes* | 2.023952668 |
| PF01882 Protein of unknown function DUF58 | *Nasutitermes* | 2.023058679 |
| PF16576 Barrel-sandwich domain of CusB or HlyD membrane fusion | *Nasutitermes* | 2.019081913 |
| PF13531 Bacterial extracellular solute binding protein | *Nasutitermes* | 2.017240838 |
| PF07859 alpha beta hydrolase fold | *Nasutitermes* | 2.004627925 |
| PF04754 Putative transposase YhgA-like | *Nasutitermes* | 2.001992129 |
| PF03328 HpcH HpaI aldolase citrate lyase family | *Nasutitermes* | 2.001831403 |
