# Supplementary material for: The tropical cookbook: Termite diet and phylogenetics—Over geographical origin—Drive the microbiome and functional genetic structure of nests
Source: Front Microbiol. 2023 Mar 14;14:1089525. doi: 10.3389/fmicb.2023.1089525 (PMC10043212; doi:10.3389/fmicb.2023.1089525)
Supplement: Supplementary file 25 [file Table_6.DOCX]

**Fig. S1.** PLS-DA scores plot using SPME-GC-TOF-MS data from the non-targeted analysis, feeding was used as a class to build the model. The same model is represented with different colouring, according to: A) feeding substrate: soil, black; wood, dark beige; B) genera: *Anoplotermes*, orange; *Cubitermes*, blue; *Nasutitermes*, brown; *Neocapritermes*, green; C) geographical location: Cameroon, blue; French Guiana, red; Malawi, yellow. Anba: *Anoplotermes banksii*; Cubin: *Cubitermes inclitus*; Cubug: *Cubitermes* aff. *ugandensis*; Cubmu: *Cubitermes muneris*; Cubpl: *Cubitermes planifrons*; Cubsu: *Cubitermes sulcifrons*; Naslu: *Nasutitermes lujae*; Nassi: *Nasutitermes similis*; Nassp*: Nasutitermes* sp.*;* Neota: *Neocapritermes taracua*; CM: Cameroon; FG: French Guiana; MW: Malawi; I: Feeding Group I; II: Feeding Group II; III: Feeding Group III; IV: Feeding Group IV.

**Fig. S2.** PLS-DA scores plot using SPME-GC-TOF-MS data from the non-targeted analysis, using the 10 most decisive compounds, different classes were used for model creation. A) Colouring according to genera: *Anoplotermes*, orange; *Cubitermes*, blue; *Nasutitermes*, brown; *Neocapritermes*, green. B) Colouring according to geographical location: Cameroon, blue; French Guiana, red; Malawi, yellow. Anba: *Anoplotermes banksii*; Cubin: *Cubitermes inclitus*; Cubug: *Cubitermes* aff. *ugandensis*; Cubmu: *Cubitermes muneris*; Cubpl: *Cubitermes planifrons*; Cubsu: *Cubitermes sulcifrons*; Naslu: *Nasutitermes lujae*; Nassi: *Nasutitermes similis*; Nassp*: Nasutitermes* sp.*;* Neota: *Neocapritermes taracua*; CM: Cameroon; FG: French Guiana; MW: Malawi; I: Feeding Group I; II: Feeding Group II; III: Feeding Group III; IV: Feeding Group IV.

**Fig. S3.** Venn diagram representation of the annotated OTUs per sample. A) Anba 1-3 versus Negative control. B) Anba 1-2 and Anba 4 versus negative control. C) Cubitermes representatives versus negative control; D) Nasutitermes representatives versus negative control.

**Fig. S4.** Barplot of PFAM interrogated terms. A) “Cellulose” retrieved terms. B) “Antibiotic” retrieved terms. Ban: *Anoplotermes banksii*; CubIn: *Cubitermes inclitus*; CubMu: *Cubitermes muneris*; CubPl: *Cubitermes planifrons*; CubSu: *Cubitermes sulcifrons*; NasLu: *Nasutitermes lujae*; Nassp*: Nasutitermes* sp.. Numbers after sample name indicate replicate number.

**Fig. S5.** Barplot of PFAM interrogated terms. A) “Phenol” retrieved terms. B) “Resistance” retrieved terms. Ban: *Anoplotermes banksii*; CubIn: *Cubitermes inclitus*; CubMu: *Cubitermes muneris*; CubPl: *Cubitermes planifrons*; CubSu: *Cubitermes sulcifrons*; NasLu: *Nasutitermes lujae*; Nassp*: Nasutitermes* sp.. Numbers after sample name indicate replicate number.

**Fig. S6.** Heatmap of genetic content profiles for the PFAM annotation. Samples or genes clustered using Euclidean distance. Ban: *Anoplotermes banksii*; CubIn: *Cubitermes inclitus*; CubMu: *Cubitermes muneris*; CubPl: *Cubitermes planifrons*; CubSu: *Cubitermes sulcifrons*; NasLu: *Nasutitermes lujae*; Nassp*: Nasutitermes* sp.. Numbers after sample name indicate replicate number.

**Fig. S7.** Volcano plot for the DPGs in the comparisons between microbial nests metagenomic samples, when using COG annotated genes as a reference. A) *Cubitermes* vs. *Anoplotermes*. B) *Nasutitermes* vs. *Cubitermes*. C) *Nasutitermes* vs *Anoplotermes*. Cub.: *Cubitermes*; Ban.: *Anoplotermes*; Nas.: *Nasutitermes*. Blue: genes with higher presence on the first component of the comparison (up-“regulated”); Red: genes with lower presence on the first component of the comparison (down-“regulated”).
